# Environmental Enrichment Enhances Visual Function in Adult Amblyopia: Association With miR‐132/HDAC3 Regulation

**DOI:** 10.1155/np/6559412

**Published:** 2026-09-08

**Authors:** Suzhen Ding, Yong Li, Yutong Zhang, Tingyu Zhang, Lan Li

**Affiliations:** ^1^ Department of Ophthalmology, The Affiliated Calmette Hospital of Kunming Medical University, Kunming 650051, Yunnan, China; ^2^ Department of Ophthalmology, Yunnan First People’s Hospital, Kunming 650032, Yunnan, China, ypfph.com

**Keywords:** adult amblyopia, enriched environment, HDAC3, miR-132, visual cortex, visual evoked potential

## Abstract

Amblyopia is a neurodevelopmental disorder for which effective treatments in adults are limited due to reduced cortical plasticity. Environmental enrichment (EE) can partially restore this plasticity, but the underlying molecular mechanisms remain unclear. This study investigated whether the miR‐132/HDAC3 pathway is associated with EE‐induced visual recovery in adult amblyopic mice. An adult amblyopia model (*n* = 64) was established via monocular deprivation (MD). Amblyopic mice were assigned to untreated, standard housing (SH), or EE groups, with normal mice as controls. Visual function was assessed using flash visual evoked potentials (fVEP), and molecular analyses of miR‐132 and HDAC3 in the primary visual cortex (V1) were performed. In vitro studies included a dual‐luciferase assay to verify miR‐132‐HDAC3 targeting and evaluated the effects of miR‐132/HDAC3 overexpression on cortical neurons. Compared to SH, EE‐reared amblyopic mice showed (1) increased N1‐P2 wave amplitude in fVEP and (2) upregulated miR‐132 and downregulated HDAC3 in V1. In vitro, HDAC3 was confirmed as a direct miR‐132 target, and miR‐132 overexpression suppressed HDAC3 while promoting structural remodeling in visual cortical neurons. These findings suggest that the miR‐132/HDAC3 pathway potentially contributes to EE‐induced visual restoration in adult amblyopia.

## 1. Introduction

Amblyopia is a neurodevelopmental visual disorder resulting from abnormal visual experience during childhood, manifesting as reduced best‐corrected visual acuity despite normal ocular anatomy. This condition impairs multiple visual functions, including contrast sensitivity and stereopsis, with significant psychosocial consequences ranging from anxiety to diminished quality of life [1, 2]. While effective treatments exist during the visual critical period (<12 years), adult amblyopia cannot be effectively treated. Elucidating its pathogenic mechanisms and developing adult‐specific interventions are, therefore, crucial research priorities.

In their seminal 1963 work, Wiesel and Hubel developed the monocular deprivation (MD) amblyopia model, demonstrating that MD renders cortical neurons unresponsive to amblyopic eye stimulation. Although the structure of the amblyopic eye and its visual pathways are intact, the corresponding cortical area cannot perceive external stimuli. The essence of this phenomenon is the weakening of synaptic plasticity in the visual cortex [3, 4]. Our previous studies established an MD mouse model, showing reduced visual function in amblyopic eyes (assessed by flash visual evoked potential [fVEP] and behavioral tests) and impaired long‐term potentiation (LTP) in V1 [5]. As LTP is one of the important manifestations of synaptic plasticity, these findings collectively underscore the crucial role of cortical plasticity deficits in amblyopia pathogenesis.

Environmental enrichment (EE) has been shown to enhance hippocampal neuroplasticity through multiple mechanisms, including modulation of postsynaptic proteins, synaptogenesis, neurogenesis, and LTP [6]. Long‐term EE exposure induces lasting changes in neuronal excitability and synaptic plasticity in adult brains [7]. Therapeutically, EE improves behavioral and cellular deficits in various neurological disorders, including Parkinson’s disease, Alzheimer’s disease, and depression [8]. However, its effects on adult amblyopia and the underlying molecular mechanisms remain poorly understood [9, 10].

EE modulates gene expression through epigenetic mechanisms involving histone modifications and noncoding RNAs (e.g., miRNAs) [6]. Notably, plasticity‐related miRNAs (miR‐132, miR‐212, and miR‐124) critically regulate synaptic function and are implicated in neurodegenerative processes [11, 12]. Histone acetylation dynamics, governed by histone acetyltransferases (HATs)/histone deacetylases (HDACs) balance, are the main driving forces for gene expression regulation. HDAC plays an important role in learning and memory, synaptic plasticity, and adult neurogenesis [13]. This study explores the association between EE and visual function in adult amblyopic mice, focusing on the involvement of the miR‐132/HDAC3 pathway. Our findings may inform novel therapeutic strategies for adult amblyopia.

## 2. Materials and Methods

### 2.1. Modeling and Grouping of Experimental Animals

Establishment of the MD adult amblyopic mouse model: 65 healthy male C57BL/6 mice aged 3 weeks were selected. The MD procedure was described previously [5]. Mice were anesthetized with pentobarbital sodium (80 mg/kg, i.p.), and complete anesthesia was confirmed by the absence of a withdrawal response to the toe or tail pinch. One eye was randomly selected for deprivation. The periocular skin was disinfected with povidone‐iodine solution, and excess hair around the eyelid margins was carefully trimmed using ophthalmic scissors. To ensure complete and sustained eyelid closure throughout the deprivation period (from 3 to 8 weeks of age), ~1 mm of the upper and lower eyelid margins was resected using ophthalmic scissors, taking care to avoid injury to the cornea. The two eyelid margins were then approximated and closed using mattress sutures with a 7–0 nonabsorbable suture. A small amount of tobramycin ophthalmic ointment was applied to the wound surface.

After surgery, mice were placed in an animal incubator (constant temperature) in a lateral position to prevent respiratory obstruction and were allowed to recover from anesthesia. Once fully awake and moving normally, they were returned to their home cages. Food and water intake were monitored daily. For the first postoperative week, tobramycin ointment was applied daily to the sutured eye, and the wound was examined for suture dehiscence or signs of infection. If any suture loosened, it was immediately resutured. Animals with persistent ocular infection, corneal opacity, or unrepaired suture breakage were excluded from the study.

At 8 weeks of age (adulthood), the sutured eyelids were reopened under light anesthesia, and fVEP was recorded to confirm the successful establishment of amblyopia. This protocol yielded 64 adult amblyopic mice that were included in subsequent experiments.

**Figure 1:**
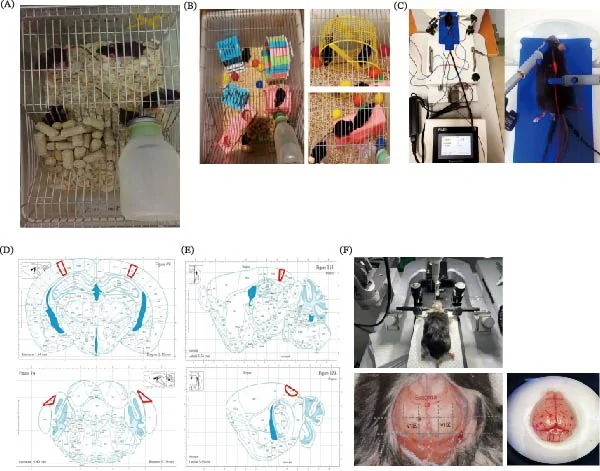
Schematic diagrams of methods used in this study. (A) Mice raised in SH. (B) Mice raised in EE. (C) fVEP recording. (D) Coronal view of stereotaxic mouse brain atlas. (E) Sagittal view of stereotaxic mouse brain atlas. (F) Visual cortex V1 tissue sampling using stereotaxic apparatus and stereomicroscope. EE, enriched environment; fVEP, flash visual evoked potentials; SH, standard housing; V1, primary visual cortex.

These amblyopic mice were randomly allocated into three experimental groups: (1) untreated amblyopia group (*n* = 20): physiological parameters in bilateral V1 visual cortex were compared between amblyopic and contralateral sides. (2) Standard housing (SH) group (*n* = 21): mice were housed in standard cages (30 × 20 × 12 cm^3^, 5 mice/cage) with bedding for 30 days (Figure 1A). (3) EE group (*n* = 23): mice were housed in large cages (60 × 40 × 30 cm^3^, 8 mice/cage) containing various toys (running wheels, building blocks, wooden structures, etc.) that were rearranged daily for 30 days (Figure 1B).

In addition, 16 healthy 3‐week‐old mice were randomly selected as normal controls for the animal experiment and subjected to SH (*n* = 8) or EE (*n* = 8) intervention. Separately, three mice aged 2 weeks were randomly selected, and their V1 tissue was collected for primary cell culture and detection.

Experimental procedures were performed in accordance with the Association for Research in Vision and Ophthalmology (ARVO) Statement for the Use of Animals in Ophthalmic and Vision Research and were approved by the Kunming Medical University Animal Care Committee (Number kmmu2018010). All efforts were made to minimize animal suffering and the number of animals used in the experiments.

### 2.2. fVEP Recording

Following Strain and Tedford’s method [14], fVEP responses were recorded using the RETI‐port/scan21 system (Roland Consult, Brandenburg, Germany) as previously described [5], with additional details provided below: mice were anesthetized with pentobarbital sodium (80 mg/kg, i.p.), and the depth of anesthesia was confirmed by the absence of a withdrawal reflex in response to a toe or tail pinch. Any mouse with corneal opacity (determined by visual inspection) was excluded from the study. The body temperature was maintained at 36.5 ± 0.5°C using a constant‐temperature blanket monitored by a rectal probe. The animal was then fixed on a recording platform in a prone position. The recording electrode was inserted subcutaneously at the midline of the scalp, exactly at the midpoint of a line connecting the anterior margins of both ears. The reference electrode was placed in the cheek pouch, and the ground electrode was inserted subcutaneously beneath the tail vein, taking care to avoid puncturing the vein. Importantly, to ensure that the recorded signals originated exclusively from the stimulated eye, the contralateral (nonstimulated) eye was completely covered with a black, light‐tight occluder (Figure 1C).

The flash stimulus was delivered from a stimulator positioned 20 cm away from the mouse. The stimulus parameters were as follows: intensity 5 dB (9.49 cd/m^2^), flash duration 10 µs, and stimulation frequency 1 Hz. For each recording, three trains of 100 artifact‐free sweeps were collected and averaged. The sampling rate was 1 kHz, and a bandpass filter of 1–50 Hz was applied. Throughout the recording session, the animal’s condition was continuously monitored.

After completion of the recording, mice were placed in an animal incubator for recovery and were returned to their home cages only after fully regaining consciousness and normal motor activity (*n* = 20–23/group).

### 2.3. Methods for Sampling Visual Cortex V1 Region of Mice

According to the stereotaxic mouse brain atlas, the primary visual cortex (V1) spans from lambda from −2.15 to −5.19 mm along the coronal plane (Figure 1D) and extends from 1.56 to 3.25 mm lateral to the midline in the sagittal plane (Figure 1E). Using lambda and the midline as anatomical reference points, we first identified the boundaries of the V1 region. Stereotaxic coordinates were then used to create fiducial marks at these boundaries. Following craniotomy and brain extraction, the V1 tissue was precisely dissected under a stereomicroscope using the fiducial marks as guides (Figure 1F). This standardized approach ensured both anatomical accuracy and sampling consistency across specimens.

### 2.4. Real‐Time Quantitative RT‐PCR

Total RNA was isolated from either cultured cells or visual cortex V1 tissue using the Trizol reagent, followed by purification with chloroform and isopropanol precipitation. The SureScript‐First‐strand‐cDNA‐synthesis kit and KR116 were used for reverse transcription of miRNA and mRNA, respectively, and cDNA was used as a template for amplification. For data normalization, miRNA expression levels were standardized against U6 snRNA, while mRNA levels were normalized to GAPDH expression (for animal experiments, *n* = 6–8/group; for cell experiments, *n* = 3/group). Primers:

**Table:** 

Target gene	Forward primer	Reverse primer
HDAC1	CTGCGTTCTATTCGCCCAGA	AAGCCATCAAACACCGGACA
HDAC2	AGGGGCTAAAGTCCGCTTGT	TAGGGCACAGAGCGGGATAG
HDAC3	GGTCTCTATAAGAAGATGAT	GATGTAGTCCTCAGAATG
miR‐124‐3p	TAAGGCACGCGGTGAATGCCAA	GTCGTATCCAGTGCAGGGT
miR‐132‐3p	TAACAGTCTACAGCCATGGTCG	GTCGTATCCAGTGCAGGGT
miR‐137‐3p	TTATTGCTTAAGAATACCGCGTAG	GTCGTATCCAGTGCAGGGT
miR‐212‐3p	ACCTTGGCTCTAGACTGCTTACT	GTCGTATCCAGTGCAGGGT
miR‐34a‐5p	TGGCAGTGTCTTAGCTGGTTGT	GTCGTATCCAGTGCAGGGT
miR‐9‐5p	TCTTTGGTTA TCTAGCTGTATGA	GTCGTATCCAGTGCAGGGT

### 2.5. Western Blotting Assay

Protein samples were lysed in RIPA buffer and quantified using the BCA protein assay. A 5% concentrated gel and 10% separating gel were prepared separately, and then, proteins were electrophoresed on SDS‐PAGE gel and transferred onto a methanol‐activated PVDF membrane. The membrane was blocked with 5% BSA, incubated with a primary antibody diluted 1:2000, and then with an HRP‐conjugated secondary antibody. Protein bands were visualized using enhanced chemiluminescence (Beyotime) and quantified with ImageJ software (*n* = 6–7/group).

### 2.6. Primary Cell Culture of Visual Cortical Neurons

After euthanizing 2‐week‐old C57 mice (*n* = 3) with cervical dislocation, the brain was removed on a clean bench. The dura mater was stripped off, and the cortical tissue was directly taken from the visual cortex region using tweezers and placed in a culture dish, washed with PBS containing 5% antibiotics. A 1.5 mL of 0.125% trypsin was added in a 60 mm culture dish, and the tissue was chopped with scissors and then digested in a 37°C incubator for 5 min. DMEM containing 10% fetal bovine serum was added to halt digestion and mixed gently. After settling for 2 min, the supernatant was filtered into another centrifuge tube, and serum‐free DMEM was added to the remaining tissue for further mixing and settling. The supernatant was collected, centrifuged at 1000 r/min for 6 min, the supernatant was discarded, and DMEM containing 20% fetal bovine serum was added. The solution was pipetted repeatedly to create a single‐cell suspension, and then, the suspension was seeded in a six‐well plate (with coverslips treated with poly‐L‐lysine placed in advance) and incubated at 37°C with 5% CO_2_. After 1 h, the six‐well plate was replaced, and after 24 h, Ara‐C was added to achieve a final concentration of 10 μmol/L to inhibit excessive proliferation of nonneuronal cells. After 24 h, half of the medium was replaced, and subsequently, medium changes were performed every other day. After cell seeding, the growth status and morphological changes of the cells were observed under an inverted phase‐contrast microscope every 24 h, and photographs were taken for records.

### 2.7. Dual Luciferase Reporter Assay

First, bioinformatics analysis was conducted to predict the possible binding sites of miR‐132. The 3^′^ UTR region of the HDAC3 gene (~500 bp) was amplified by PCR and cloned into the pGL3‐promoter vector to construct the wild‐type firefly luciferase plasmid (HDAC3‐WT). At the same time, primers were designed to introduce mutations at the predicted miR‐132 binding sites to construct the mutant firefly luciferase plasmid (HDAC3‐MUT). The SH‐SY5Y cell line was transfected using Lipofectamine 3000 reagent and grouped as follows: (1) firefly plasmid pGL3‐HDAC3‐WT + miR‐132 mimics group; (2) firefly plasmid pGL3‐HDAC3‐WT + mimics NC group; (3) firefly plasmid pGL3‐HDAC3‐MUT + miR‐132 mimics group; (4) firefly plasmid pGL3‐HDAC3‐MUT + mimics NC group. Each group was cotransfected with a Renilla luciferase fluorescence plasmid as an internal reference. After 48 h of transfection, cells were collected, and the activities of firefly luciferase and Renilla luciferase were detected using the Dual‐Luciferase Reporter Assay System (Promega). The activity of Renilla luciferase was used to normalize the activity of firefly luciferase, and relative luciferase activity was calculated (*n* = 3/group).

### 2.8. Scanning Electron Microscopy (SEM)

The morphological alterations of neurons in the mouse visual cortex were analyzed using SEM. Briefly, neuronal samples were sequentially fixed in 4% glutaraldehyde and 1% osmium tetroxide (1 h each), followed by graded ethanol dehydration (50%, 70%, 90%, 95%, and 100%; 10 min per step). Critical‐point drying was then performed to preserve ultrastructural integrity. Subsequently, samples were sputter‐coated with a thin conductive layer using a vacuum coater. Neuronal morphology was finally examined under SEM (*n* = 3/group).

### 2.9. Immunofluorescence

Cells grown on coverslips were washed three times with PBS, fixed with 4% paraformaldehyde for 30 min at room temperature, and rinsed with 0.01 M PBS. Permeabilization was then carried out using 0.2% Triton X‐100 in PBS for 20 min at room temperature, followed by another PBS rinse. After blocking with 5% normal goat serum for 60 min at room temperature, the samples were incubated with the primary antibody at 4°C overnight. Subsequent steps included four PBST washes, incubation with fluorescent secondary antibody for 2 h at room temperature, four additional PBST washes, and final mounting with DAPI‐containing antifade mounting medium for fluorescence microscopy imaging (*n* = 3/group).

### 2.10. Statistical Analysis

All data were analyzed using SPSS (26.0, IBM) and GraphPad Prism (9.5.0, GraphPad Software) and are presented as mean ± SEM. Normality and homogeneity of variance were assessed using the Shapiro–Wilk and Levene’s tests, respectively. Two‐group comparisons were performed using unpaired or paired *t*‐tests as appropriate. For multiple groups, one‐way ANOVA with Fisher’s least significant difference (LSD) post hoc test was used when variances were homogeneous; otherwise, Welch’s ANOVA with the Games‐Howell post hoc test was applied. Statistical significance was set at  ^∗^ *p*  < 0.05,  ^∗∗^ *p*  < 0.01,  ^∗∗∗^ *p*  < 0.001, and  ^∗∗∗∗^ *p*  < 0.0001. Detailed statistical results are provided in the Supporting Information 1.

For post hoc comparisons following ANOVA, Fisher’s LSD test was used for preplanned pairwise comparisons. This approach was chosen because comparisons were limited and prespecified based on our experimental hypotheses. Notably, the critical differences reported (e.g., miR‐132 mimic vs. control in luciferase assays) reached *p* < 0.0001, and would remain significant even under more conservative correction procedures.

### 2.11. Randomization and Blinding

Animals were randomly assigned to experimental groups using a random number table generated from a standard statistical textbook. The randomization procedure was performed by an investigator who was not involved in any subsequent experimental procedures or data analyses. The investigator who performed fVEP recordings, tissue sampling, tissue processing for electron microscopy, image acquisition, Western blot quantification, and statistical analysis was blinded to the group allocation throughout the experiment and during data analysis. Blinding was maintained until all the data were finalized.

### 2.12. Replicates and Sample Size

In this study, *n* represents the number of biologically independent samples: for animal studies, one animal = one biological replicate; for cell studies, one biological replicate = one independent cell culture passage. Technical replicates were not used to define *n*. This definition applies to all figures and statistical analyses.

Of note, fVEP recordings were performed on all animals before tissue collection, which is why the sample size for fVEP (*n* = 20–23/group) is larger than that for molecular or morphological assays (*n* = 6–8 or *n* = 3, respectively).

## 3. Results

### 3.1. The Effect of EE on Visual Function in Adult Amblyopic Mice

**Figure 2:**
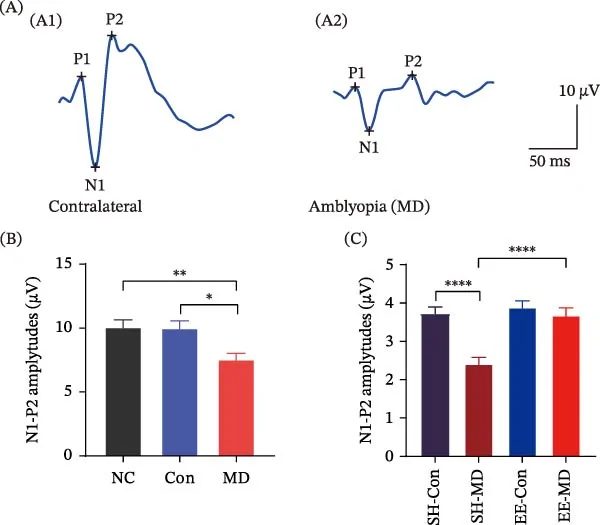
fVEP amplitudes were significantly reduced in adult amblyopic mice. (A) Representative fVEP response recorded from an MD adult amblyopic mouse. The subpart A1 shows the waveform of the nondeprived eye (contralateral eye), and A2 shows the waveform of the MD eye (amblyopic eye). (B) Comparison of N1‐P2 wave amplitudes of NC eyes, contralateral eyes and amblyopic eyes (*n* = 20 animals per group [each animal = one biological replicate]). (C) Comparison of N1‐P2 wave amplitudes between contralateral eyes and amblyopic eyes of adult amblyopic mice raised in SH and EE (*n* = 21–23 animals per group [each animal = one biological replicate]).  ^∗^ *p*  < 0.05;  ^∗∗^ *p*  < 0.01;  ^∗∗∗∗^ *p*  < 0.0001. Con, contralateral; EE, enriched environment; fVEP, flash visual evoked potentials; MD, monocular deprivation; NC, normal control; SH, standard housing.

fVEP provides an objective assessment of visual function. In our MD model, 3‐week‐old mice underwent unilateral eye suturing until adulthood. fVEP recordings demonstrated significantly reduced N1‐P2 amplitudes in deprived eyes compared to both contralateral eyes (*p*  < 0.05) and normal controls (*p*  < 0.01; Figure 2B), confirming successful establishment of adult amblyopia. Following 30 days of housing in either SH or EE, EE‐reared mice showed significantly greater N1‐P2 amplitudes in amblyopic eyes versus SH (*p*  < 0.001), with responses approaching those of contralateral eyes (Figure 2C). These results suggest that EE enhances the visual function in adult amblyopic mice.

### 3.2. The Effect of EE on Expression of miR‐132 and HDAC3 in Visual Cortex of Adult Amblyopic Mice

**Figure 3:**
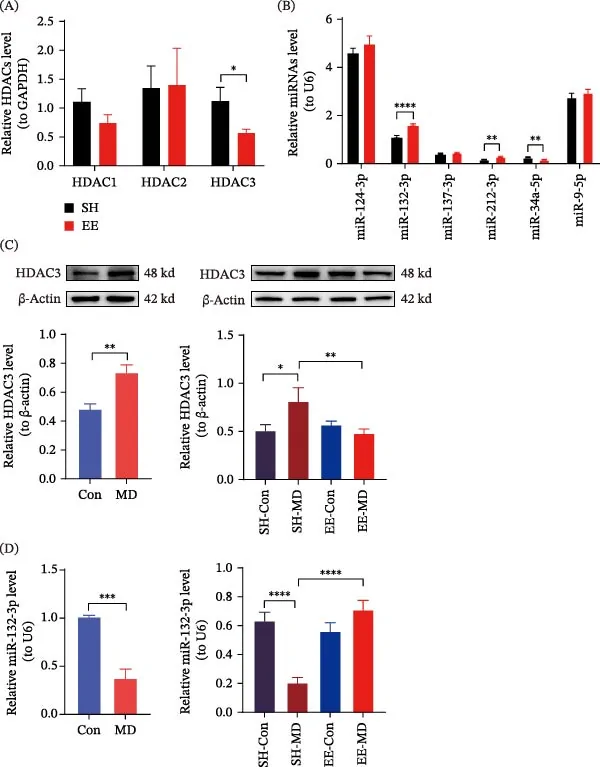
EE exposure is associated with epigenetic changes and amelioration of amblyopic deficits. (A) Differential expression of HDACs in visual cortex of normal mice raised in SH and EE (*n* = 8 animals per group [each animal = one biological replicate]). (B) Differential expression of miRNAs in visual cortex of normal mice raised in SH and EE (*n* = 6 animals per group [each animal = one biological replicate]). (C) Expression of HDAC3 in bilateral V1 regions of adult amblyopic mice (*n* = 6 animals per group); comparison of HDAC3 after being raised in EE and SH (*n* = 7 animals per group). (D) Expression of miR‐132 in bilateral V1 regions of adult amblyopic mice (*n* = 6 animals per group); comparison of miR‐132 after being raised in SH and EE (*n* = 15–17 animals per group).  ^∗^ *p*  < 0.05;  ^∗∗^ *p*  < 0.01;  ^∗∗∗^ *p*  < 0.001;  ^∗∗∗∗^ *p*  < 0.0001. Con, contralateral; EE, enriched environment; HDAC, histone deacetylase; MD, monocular deprivation; SH, standard housing; V1, primary visual cortex.

To investigate how EE enhances visual function, we examined epigenetic markers linked to EE exposure, especially those involved in activity‐dependent plasticity [6, 15, 16]. In normal mice, EE induced distinct epigenetic modifications, revealing specific molecular responses. Compared to SH, EE exposure specifically reduced HDAC3 expression (*p*  < 0.05) without significantly affecting HDAC1/2 levels (Figure 3A). Concurrently, EE significantly upregulated miR‐132 (*p*  < 0.0001) and miR‐212 (*p*  < 0.01) while downregulating miR‐34a (*p*  < 0.01), with no effect on other neural development‐related miRNAs (Figure 3B). In adult amblyopic mice, we observed an epigenetic imbalance in the amblyopic V1 region, characterized by elevated HDAC3 (*p*  < 0.01) and reduced miR‐132 (*p*  < 0.001) compared to contralateral sides (Figure 3C,D). EE intervention was associated with a reversal of these abnormalities, normalizing HDAC3 expression to contralateral levels (*p*  < 0.01, Figure 3C) and elevating miR‐132 beyond both SH (*p*  < 0.0001) and contralateral values (Figure 3D). These findings are consistent with the idea that EE is associated with the restoration of molecular homeostasis in the adult amblyopic visual cortex, with a potential link to selective regulation of the HDAC3/miR‐132 axis.

### 3.3. HDAC3 Is a Target Gene of miR‐132

**Figure 4:**
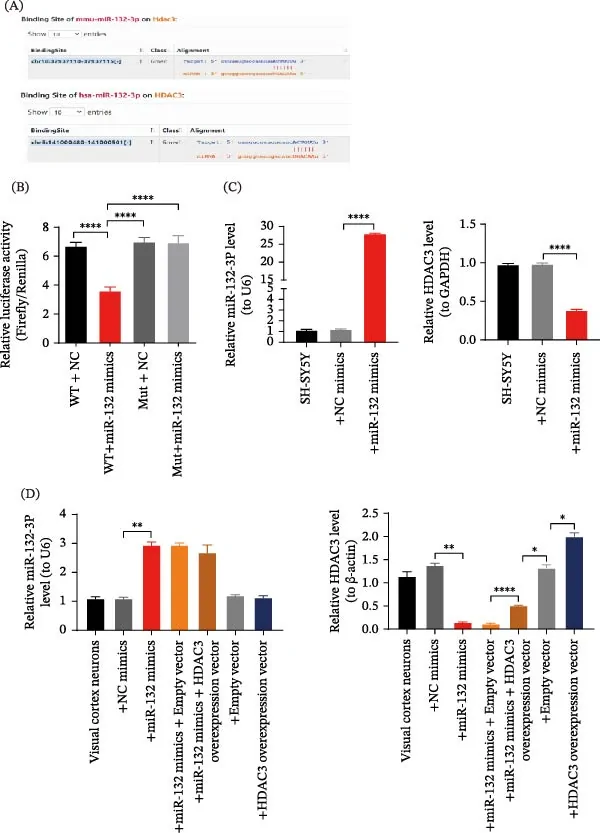
miR‐132 directly targets the HDAC3 3^′^UTR (Luciferase assays in SH‐SY5Y cells were used for biochemical target validation; primary neuron cultures addressed visual cortex mechanisms). (A) The predicted binding site of miR‐132‐3p and HDAC3 mRNA in mouse and human. (B) Result of dual‐luciferase reporter assays. *n* = 3 independent biological replicates (each replicate from a separate cell passage). (C) miR‐132 levels and HDAC3 expression in SH‐SY5Y cells following miR‐132 mimic transfection (RT‐PCR). *n* = 3 independent biological replicates (each from a separate cell passage). (D) miR‐132 expression and HDAC3 expression in visual cortex neurons with combined miR‐132 mimic/HDAC3 overexpression adenovirus transfection (RT‐PCR). *n* = 3 independent biological replicates (each from a separate primary neuron culture preparation).  ^∗^ *p*  < 0.05;  ^∗∗^ *p*  < 0.01;  ^∗∗∗∗^ *p*  < 0.0001. HDAC, histone deacetylase; Mut, mutant; NC, normal control; RT‐PCR, reverse transcription polymerase chain reaction; WT, wild type.

Bioinformatics analysis revealed a complementary sequence between HDAC3 and miR‐132‐3p (Figure 4A). To validate this targeting relationship, we performed dual‐luciferase reporter assays using SH‐SY5Y cells. Normalized firefly luciferase (Fluc/Rluc) measurements demonstrated that miR‐132 significantly suppressed WT HDAC3 3^′^‐UTR activity (*p*  < 0.0001), while mutations in the miR‐132 binding sites abolished this effect (Figure 4B), confirming direct targeting.

Further experiments in SH‐SY5Y cells showed that miR‐132 overexpression markedly reduced HDAC3 expression (*p*  < 0.0001, Figure 4C). In primary mouse visual cortex neurons, cotransfection with miR‐132 mimics and HDAC3‐overexpressing adenovirus revealed that miR‐132 negatively regulated HDAC3 (*p*  < 0.01, Figure 4D). Together with the luciferase assay, these findings suggest that miR‐132 is a negative regulator of HDAC3 in visual cortex neurons.

We note that the luciferase assay was performed in SH‐SY5Y cells for biochemical validation; the relevance to visual cortex neurons was further supported by primary neuron experiments (Figure 4D) and in vivo data (Figure 3C,D).

### 3.4. Epigenetic Regulation Has a Significant Impact on Neuronal Morphology in the Visual Cortex

**Figure 5:**
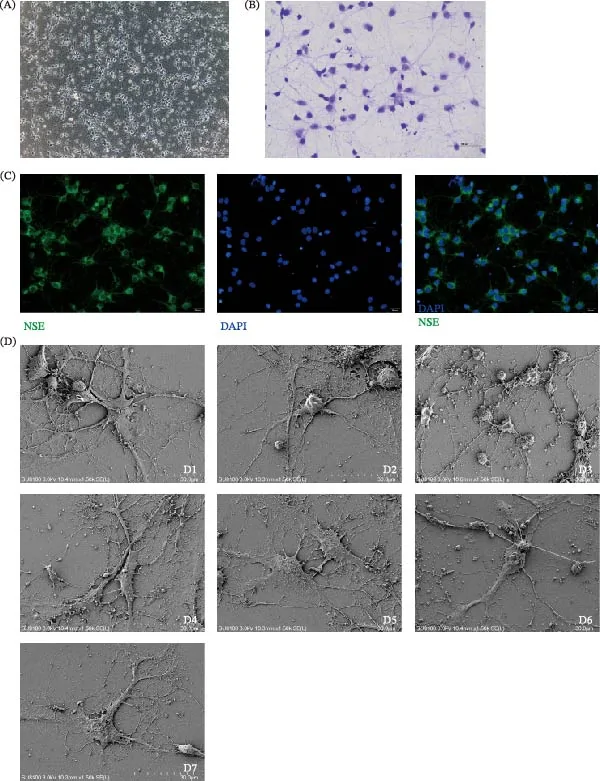
Epigenetic regulation is associated with altered morphology of visual cortex neurons. *n* = 3 independent primary neuron culture preparations (each from a different animal). (A) Phase‐contrast image of visual cortex neuron cell culture on Day 7 (100x). (B) Nissl staining image of Nissl bodies in the cytoplasm of visual cortex neurons (40x). (C) Immunofluorescence detection of the distribution of NSE in visual cortex neurons (40x). (D) Scanning electron microscopy (SEM) images of visual cortex neurons (scale bar = 30 μm, grouped according to Figure 4D): (D1, D2, D6) control neurons: intact morphology with plump somata and clearly identifiable axons and dendrites; (D3, D4) miR‐132 overexpression: increased neuronal density, abundant axons, elongated neurites, and structural features suggestive of increased interneuronal contacts (a dense mesh‐like network of processes); (D5) co‐overexpression of miR‐132 and HDAC3: intact neuronal morphology but reduced cell density and fewer interneuronal structural elements; (D7) HDAC3 overexpression alone: shrunken somata, shortened neurites, and markedly diminished structural features. HDAC, histone deacetylase; NSE, neuron‐specific enolase.

In primary mouse visual cortex neurons (identified by Nissl staining and NSE immunofluorescence, Figure 5A–C), SEM analysis revealed distinct morphological changes: control neurons exhibited intact morphology with plump somata and clearly identifiable axons and dendrites (Figure 5D [D1, D2, D6]). miR‐132 overexpression significantly increased neuronal density, accompanied by abundant axonal projections, elongated neurites, and structural features suggestive of increased interneuronal contacts (Figure 5D [D3, D4]). Co‐overexpression of miR‐132 and HDAC3 reduced both neuronal density and these structural features (Figure 5D [D5]). In contrast, HDAC3 overexpression alone induced somatic shrinkage, neurite retraction, and markedly diminished such structural features (Figure 5D [D7]). These observations indicate that miR‐132 upregulation was associated with increased density of structural elements, while HDAC3 overexpression was associated with the opposite effect, suggesting a potential interplay influencing the synaptic morphology.

**Figure 6:**
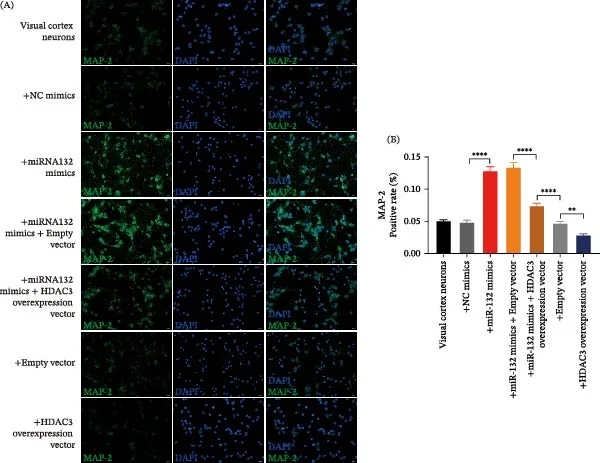
Epigenetic regulation is associated with altered MAP‐2 immunoreactivity of visual cortex neurons (40x) (*n* = 3 independent primary neuron culture preparations [each from a different animal]). (A) Effect of combined miR‐132 overexpression and HDAC3 overexpression on MAP‐2 immunoreactivity in visual cortex neurons. (B) miR‐132 overexpression was associated with increased MAP‐2 immunoreactivity, whereas HDAC3 overexpression was associated with reduced MAP‐2 immunoreactivity ( ^∗∗^ *p*  < 0.01;  ^∗∗∗∗^ *p*  < 0.0001). HDAC, histone deacetylase; MAP‐2, microtubule‐associated protein 2.

Microtubule‐associated protein 2 (MAP‐2), a neuron‐specific cytoskeletal protein, regulates neuronal growth, differentiation, and plasticity and is implicated in LTP [17, 18]. Immunofluorescence analysis of MAP‐2 expression in mouse visual cortex neurons demonstrated that miR‐132 overexpression significantly upregulated MAP‐2 levels (*p*  < 0.0001), whereas HDAC3 overexpression markedly reduced MAP‐2 expression (*p*  < 0.01) (Figure 6). This may be one of the factors contributing to the observed association between the miR‐132/HDAC3 axis and the neuronal morphology in the visual cortex.

Given the limited sample size (*n* = 3 per group) for SEM and MAP‐2 analyses, these results should be interpreted as preliminary; future studies with larger cohorts are needed to confirm these morphological observations.

Taken together, our study suggests an association between the miR‐132/HDAC3 pathway and EE‐induced visual restoration. In vitro experiments demonstrate that miR‐132 directly inhibits HDAC3, while in vivo findings associate EE‐induced visual functional improvements in adult amblyopic mice with upregulation of miR‐132 and downregulation of HDAC3. These findings are consistent with a potential involvement of this pathway.

## 4. Discussion

The MD amblyopia model represents a well‐established paradigm for investigating visual cortical plasticity [3]. By suturing eyelids during the visual developmental period, this model effectively mimics human amblyopia pathogenesis through the disruption of synaptic transmission and functional connectivity in visual cortex neurons during the critical developmental window. MD induces significant neuronal alterations, including reduced dendritic spine density in the deprived‐eye visual cortex as early as postnatal Day 28 [19]. While robust synaptic plasticity enables functional recovery during developmental critical periods, this capacity declines markedly in adulthood, contributing to the therapeutic resistance of adult amblyopia [9]. Synaptic plasticity—the activity‐dependent modulation of interneuronal connection strength—reflects the nervous system’s adaptive capacity to environmental changes or injury, measurable through electrophysiological, morphological, and molecular approaches. Notably, recent evidence demonstrates retained plasticity in the mature visual system [20, 21]. EE has been shown to reactivate ocular dominance plasticity and restore visual function in adult amblyopic animals [9], positioning EE as a promising therapeutic strategy for adult amblyopia intervention.

This study established an adult amblyopic mouse model and found that the amplitude of fVEP N1‐P2 in amblyopic eyes decreased, indicating a decline in visual function. Exposure to EE was associated with restoration of fVEP amplitude, which is an indicator for evaluating visual function and visual cortex excitability [21]. Mechanistically, HDAC3 is a direct target of miR‐132; EE may upregulate miR‐132, which in turn may inhibit HDAC3, and this cascade is associated with synaptic development in visual cortex neurons, potentially contributing to improved visual function in amblyopia. These results, combining in vitro evidence of direct regulation with in vivo correlational findings, suggest that the miR‐132/HDAC3 axis is associated with EE‐induced visual restoration in adult amblyopic mice, although direct in vivo causality requires further investigation.

EE is an experimental model that enhances brain plasticity by providing animals with complex sensory stimulation and opportunities for voluntary exercise. This approach typically involves housing mice in large cages containing various colorful objects to increase sensory input and social interactions, along with running wheels to promote physical activity. Both environmental complexity and voluntary exercise have been shown to induce similar neuroplastic changes, including increased neurogenesis, elevated neurotrophic factor levels, enhanced neurotransmitter production, and improved synaptic plasticity and cognitive function [22]. While EE has been widely used to study learning, memory, and neurological disorders, with well‐documented effects on brain regions like the hippocampus, amygdala, and prefrontal cortex [8, 22–24], its impact on the visual system has received less attention. Emerging evidence suggests EE can improve visual acuity in amblyopic eyes and restore depth perception [10, 25–27], potentially through mechanisms involving reduced GABAergic inhibition, reorganization of ocular dominance columns [9], and enhanced glucose metabolism in the visual cortex [28]. Our study builds on this evidence by identifying a regulatory pathway—the miR‐132/HDAC3 pathway—that may be associated with the visual functional improvements induced by EE in adult amblyopia, offering new therapeutic possibilities for this challenging condition. A major limitation of this study is that the EE paradigm is multifactorial, combining larger cages, increased social density, running wheels, novel objects, and physical activity. We did not dissociate the individual components of the EE. Therefore, we cannot determine which specific aspect of EE—whether physical exercise, sensory novelty, social interaction, or their combination—is most closely associated with the observed upregulation of miR‐132 and downregulation of HDAC3. This is a critical caveat because the observed effects on visual function and molecular changes could be attributable to any one or a combination of these factors. Future studies employing a component‐by‐component dissection (e.g., running wheel alone, social housing alone, or enriched objects alone) are warranted to identify the active elements that may drive the activation of the miR‐132/HDAC3 pathway and subsequent visual recovery.

miRNAs are small noncoding RNAs that posttranscriptionally regulate gene expression by binding target mRNAs to inhibit translation or promote degradation. Studies have shown that miRNAs can mediate epigenetic gene expression and are considered part of the epigenetic mechanisms [29]. Among these, miR‐132 is highly conserved across species (including humans, rodents, and primates) and abundantly expressed in the brain, where it regulates cognitive function, neurodevelopment, and synaptic plasticity [30]. Mounting evidence implicates miR‐132 in neurodegenerative disorders including Alzheimer’s disease, mild cognitive impairment, and depression [12, 31, 32]. Notably, miR‐132 shows particularly pronounced alterations in the visual cortex of MD models [33]. MD induces rapid epigenetic changes, including reduced histone H3 acetylation and decreased pri‐miR‐132/miR‐132 expression within 3 days, coinciding with ocular dominance column shifts toward the nondeprived eye and impaired synaptic plasticity in the deprived visual cortex [34]. Crucially, these plasticity deficits are prevented by miR‐132 mimic administration [34]. Our findings corroborate these observations: adult MD mice exhibited significantly reduced miR‐132 expression in the amblyopic visual cortex, while EE was associated with restored miR‐132 levels. Collectively, these studies suggest that miR‐132 plays a role in visual cortical plasticity.

Epigenetics investigates heritable gene expression changes without DNA sequence alterations, with histone modifications—particularly acetylation/deacetylation mediated by HAT and HDAC—playing crucial regulatory roles. As the most developed epigenetic drugs, HDAC inhibitors show therapeutic promise for neurological disorders by enhancing synaptic plasticity and memory [35–39]. Among HDAC classes, Class I members (HDAC1−3,8) are established memory formation inhibitors [29]. Studies demonstrate that light exposure after dark adaptation increases H3/H4 acetylation, and the Class I inhibitor TSA restores adult visual cortical plasticity [40]. EE upregulates miR‐132 to suppress HDAC3 and enhance LTP in the hippocampus of mice [41]. These results are consistent with our observation that EE is associated with the inhibition of HDAC3 expression in the visual cortex (V1) and with the upregulation of miR‐132, which may be linked to improving visual function in adult amblyopia. We acknowledge that our study lacks direct evidence of chromatin modification. Although HDAC3 is a HDAC and miR‐132 can influence transcriptional regulation, we did not measure histone acetylation or perform chromatin immunoprecipitation. Therefore, we refer to the miR‐132/HDAC3 axis as a regulatory pathway rather than a proven epigenetic mechanism. Future studies using ChIP‐qPCR or ATAC‐seq are needed to directly assess chromatin remodeling.

In summary, this study suggests an association between the EE‐associated miR‐132/HDAC3 pathway and the restoration of visual function in adult amblyopic mice, advancing our understanding of visual cortical plasticity regulation. However, three main limitations should be acknowledged. First, while our in vitro experiments demonstrate a direct regulatory relationship from miR‐132 to HDAC3, the in vivo findings remain primarily correlational as EE is a multifaceted intervention that may affect multiple molecular pathways beyond this axis. Therefore, we cannot conclude that the miR‐132/HDAC3 axis is necessary or sufficient for EE‐induced functional recovery. Future studies employing targeted manipulations—such as miR‐132 knockdown or HDAC3 overexpression in the visual cortex during EE exposure—are required to establish in vivo causality. Second, our in vitro morphological data (e.g., MAP‐2 immunostaining and SEM) indicate enhanced structural plasticity upon miR‐132 overexpression, but they do not directly demonstrate functional synaptic changes. Future studies employing electrophysiological recordings (e.g., whole‐cell patch‐clamp or multielectrode array) are needed to determine whether the observed structural remodeling translates into altered synaptic transmission or network activity. Third, although fVEP recordings provide an objective measure of cortical visual responses, they do not fully capture perceptual recovery such as visual acuity, contrast sensitivity, or binocular function. Amblyopia is a perceptual disorder, and the absence of behavioral testing (e.g., optomotor reflex or visual water maze) limits our ability to claim comprehensive visual recovery. Further studies incorporating behavioral paradigms are required to determine the functional relevance of the EE‐induced fVEP improvements. Despite these limitations, our findings identify novel molecular targets for adult amblyopia and open new avenues for developing therapeutic strategies, with the ultimate goal of translating these discoveries into clinical interventions.

## Author Contributions

**Suzhen Ding**: writing – original draft, methodology, formal analysis, investigation, conceptualization. **Yong Li**: methodology, project administration, funding acquisition. **Yutong Zhang**: validation, formal analysis, investigation. **Tingyu Zhang**: formal analysis, resources, writing – review and editing. **Lan Li**: conceptualization, methodology, project administration, funding acquisition.

## Funding

This study was supported by the National Natural Science Foundation of China (Grant 81860180).

## Disclosure

After using Deepseek, the authors reviewed and edited the content as needed and took full responsibility for the content of the published article.

## Conflicts of Interest

The authors declare no conflicts of interest.

## Supporting Information

Additional supporting information can be found online in the Supporting Information section.

## Supporting information


**Supporting Information** Table S1: Detailed statistical analyses for all experiments, including test statistics, degrees of freedom, and *p* values for each comparison.

## Data Availability

The data that support the findings of this study are available from the corresponding author upon reasonable request.
