# Supplementary material for: Environmental Enrichment Enhances Visual Function in Adult Amblyopia: Association With miR‐132/HDAC3 Regulation
Source: Neural Plast. 2026 Sep 8;2026:6559412. doi: 10.1155/np/6559412 (PMC13554963; doi:10.1155/np/6559412)
Supplement: Supplementary file 1 [file NP-2026-6559412-s001.docx]

**Statistical tables**

**Figure 2B: one-way ANOVA**

| **ANOVA** | | | | | |
| --- | --- | --- | --- | --- | --- |
| Amplytudes | | | | | |
|  | Sum of Squares | df | Mean Square | F | Sig. |
| Between Groups | 93.069 | 2 | 46.534 | 5.302 | **.007** |
| Within Groups | 675.761 | 77 | 8.776 |  |  |
| Total | 768.830 | 79 |  |  |  |

| **Multiple Comparisons** | | | | | | |
| --- | --- | --- | --- | --- | --- | --- |
| Dependent Variable: Amplytudes | | | | | | |
| LSD | | | | | | |
| (I) Group | (J) Group | Mean Difference (I-J) | Std. Error | Sig. | 95% Confidence Interval | |
|  |  |  |  |  | Lower Bound | Upper Bound |
| NC | Con | .073291666625000 | .811300811194373 | .928 | -1.542214639647630 | 1.688797972897630 |
|  | MD | 2.514375000124997^*^ | .811300811194373 | .003 | .898868693852367 | 4.129881306397628 |
| Con | NC | -.073291666625000 | .811300811194373 | **.928** | -1.688797972897630 | 1.542214639647630 |
|  | MD | 2.441083333499998^*^ | .936809483473666 | .011 | .575657331891916 | 4.306509335108079 |
| MD | NC | -2.514375000124997^*^ | .811300811194373 | **.003** | -4.129881306397627 | -.898868693852367 |
|  | Con | -2.441083333499997^*^ | .936809483473666 | **.011** | -4.306509335108079 | -.575657331891916 |
| *. The mean difference is significant at the 0.05 level. | | | | | | |

**Results:** The data were normally distributed (p > 0.05) with homogeneous variance (Levene's F(2,77) = 3.031, p= 0.054), meeting the assumptions for ANOVA. One-way ANOVA revealed a significant difference in N1-P2 amplitude among the three groups (NC: 10.08 ± 0.55; Con: 10.01 ± 0.55; MD: 7.57 ± 0.46), F(2, 77) = 5.302, p = 0.007.

Post-hoc LSD tests showed that the MD group had significantly lower N1-P2 amplitude than the NC group (mean difference = -2.51, p = 0.003, 95% CI [-4.13, -0.90]) and than the Con group (mean difference = -2.44, p = 0.011, 95% CI [-4.31, -0.58]), while no significant difference was found between the Con and NC groups (mean difference = -0.07, p = 0.928, 95% CI [-1.69, 1.54]).

**Figure 2C: one-way ANOVA**

| **ANOVA** | | | | | |
| --- | --- | --- | --- | --- | --- |
| Amplytudes | | | | | |
|  | Sum of Squares | df | Mean Square | F | Sig. |
| Between Groups | 29.649 | 3 | 9.883 | 14.911 | **.000** |
| Within Groups | 55.674 | 84 | .663 |  |  |
| Total | 85.323 | 87 |  |  |  |

| **Multiple Comparisons** | | | | | | |
| --- | --- | --- | --- | --- | --- | --- |
| Dependent Variable: Amplytudes | | | | | | |
| LSD | | | | | | |
| (I) Group | (J) Group | Mean Difference (I-J) | Std. Error | Sig. | 95% Confidence Interval | |
|  |  |  |  |  | Lower Bound | Upper Bound |
| SH-Con | SH-MD | 1.313862619047620^*^ | .251242492749212 | .000 | .814239369211566 | 1.813485868883673 |
|  | EE-Con | -.156389481710145 | .245720005342103 | .526 | -.645030659702672 | .332251696282383 |
|  | EE-MD | .053491122811594 | .245720005342103 | .828 | -.435150055180933 | .542132300804122 |
| SH-MD | SH-Con | -1.313862619047620^*^ | .251242492749212 | **.000** | -1.813485868883673 | -.814239369211566 |
|  | EE-Con | -1.470252100757764^*^ | .245720005342103 | .000 | -1.958893278750291 | -.981610922765237 |
|  | EE-MD | -1.260371496236025^*^ | .245720005342103 | .000 | -1.749012674228552 | -.771730318243498 |
| EE-Con | SH-Con | .156389481710145 | .245720005342103 | .526 | -.332251696282383 | .645030659702672 |
|  | SH-MD | 1.470252100757764^*^ | .245720005342103 | .000 | .981610922765237 | 1.958893278750292 |
|  | EE-MD | .209880604521739 | .240070514407347 | .384 | -.267525941184444 | .687287150227922 |
| EE-MD | SH-Con | -.053491122811594 | .245720005342103 | .828 | -.542132300804122 | .435150055180933 |
|  | SH-MD | 1.260371496236025^*^ | .245720005342103 | **.000** | .771730318243498 | 1.749012674228553 |
|  | EE-Con | -.209880604521739 | .240070514407347 | **.384** | -.687287150227922 | .267525941184444 |
| *. The mean difference is significant at the 0.05 level. | | | | | | |

**Results:** The data were normally distributed (p > 0.05) with homogeneous variance (Levene's F(3, 84)= 0.376, p= 0.771), meeting the assumptions for ANOVA. A one‑way ANOVA revealed a significant difference in N1‑P2 amplitude among the four groups (SH‑Con: 3.73 ± 0.16; SH‑MD: 2.42 ± 0.16; EE‑Con: 3.89 ± 0.16; EE‑MD: 3.68 ± 0.20), F(3, 84) = 14.911, p < 0.0001.

Post‑hoc LSD tests showed that the SH‑MD group had significantly lower N1‑P2 amplitude than the SH‑Con group (mean difference = –1.31, p < 0.0001, 95% CI [–1.81, –0.81]), and the EE‑MD group had significantly higher amplitude than the SH‑MD group (mean difference = 1.26, p < 0.0001, 95% CI [0.77, 1.75]). No significant difference was observed between the EE‑MD and EE‑Con groups (mean difference = –0.21, p = 0.384, 95% CI [–0.69, 0.27]).

**Figure 3A: Independent sample t-test**

**①HDAC1**

| **Independent Samples Test** | | | | | | | | | | |
| --- | --- | --- | --- | --- | --- | --- | --- | --- | --- | --- |
|  | | Levene's Test for Equality of Variances | | t-test for Equality of Means | | | | | | |
|  |  | F | Sig. | t | df | Sig. (2-tailed) | Mean Difference | Std. Error Difference | 95% Confidence Interval of the Difference | |
|  |  |  |  |  |  |  |  |  | Lower | Upper |
| HDAC1 | Equal variances assumed | 1.852 | **.195** | -1.496 | 14 | **.157** | -.369122104500000 | .246755736973578 | -.898360524328273 | .160116315328273 |
|  | Equal variances not assumed |  |  | -1.496 | 11.623 | .161 | -.369122104500000 | .246755736973578 | -.908699710730309 | .170455501730309 |

**②HDAC2**

| **Independent Samples Test** | | | | | | | | | | |
| --- | --- | --- | --- | --- | --- | --- | --- | --- | --- | --- |
|  | | Levene's Test for Equality of Variances | | t-test for Equality of Means | | | | | | |
|  |  | F | Sig. | t | df | Sig. (2-tailed) | Mean Difference | Std. Error Difference | 95% Confidence Interval of the Difference | |
|  |  |  |  |  |  |  |  |  | Lower | Upper |
| HDAC2 | Equal variances assumed | .680 | **.423** | .069 | 14 | **.946** | .049521124750000 | .720227429893020 | -1.495213079157793 | 1.594255328657794 |
|  | Equal variances not assumed |  |  | .069 | 11.249 | .946 | .049521124750000 | .720227429893020 | -1.531415423634283 | 1.630457673134284 |

**③HDAC3**

| **Independent Samples Test** | | | | | | | | | | |
| --- | --- | --- | --- | --- | --- | --- | --- | --- | --- | --- |
|  | | Levene's Test for Equality of Variances | | t-test for Equality of Means | | | | | | |
|  |  | F | Sig. | t | df | Sig. (2-tailed) | Mean Difference | Std. Error Difference | 95% Confidence Interval of the Difference | |
|  |  |  |  |  |  |  |  |  | Lower | Upper |
| HDAC3 | Equal variances assumed | 27.059 | **.000** | -2.437 | 14 | .029 | -.552092257875000 | .226507148220448 | -1.037901774096438 | -.066282741653562 |
|  | Equal variances not assumed |  |  | -2.437 | 7.632 | **.042** | -.552092257875000 | .226507148220448 | -1.078833142435245 | -.025351373314755 |

**Results**: HDAC1: The data were normally distributed (p > 0.05) with homogeneous variance (p = 0.195). An independent‑samples t‑test revealed no significant difference between the EE group (0.76 ± 0.13) and the SH group (1.17 ± 0.21), t(14) = –1.495, p = 0.157, 95% CI [–0.90, 0.16].

HDAC2: The data were normally distributed (p > 0.05) with homogeneous variance (p = 0.423). An independent‑samples t‑test revealed no significant difference between the EE group (1.41 ± 0.62) and the SH group (1.37 ± 0.36), t(14) = 0.069, p = 0.946, 95% CI [–1.50, 1.59].

HDAC3: The data were normally distributed (p > 0.05) but with unequal variance (p < 0.0001). A Welch‑corrected t‑test showed that the EE group (0.59 ± 0.05) had significantly lower HDAC3 levels than the SH group (1.14 ± 0.22), t(7.632) = –2.437, p = 0.042, 95% CI [–1.08, –0.03].

**Figure 3A: Independent sample t-test**

**①miR-124**

| **Independent Samples Test** | | | | | | | | | | |
| --- | --- | --- | --- | --- | --- | --- | --- | --- | --- | --- |
|  | | Levene's Test for Equality of Variances | | t-test for Equality of Means | | | | | | |
|  |  | F | Sig. | t | df | Sig. (2-tailed) | Mean Difference | Std. Error Difference | 95% Confidence Interval of the Difference | |
|  |  |  |  |  |  |  |  |  | Lower | Upper |
| miR124 | Equal variances assumed | .515 | **.490** | 1.043 | 10 | **.321** | .386797066333333 | .370790623213387 | -.439375927200618 | 1.212970059867284 |
|  | Equal variances not assumed |  |  | 1.043 | 8.162 | .327 | .386797066333333 | .370790623213387 | -.465297838663782 | 1.238891971330448 |

**②miR-132**

| **Independent Samples Test** | | | | | | | | | | |
| --- | --- | --- | --- | --- | --- | --- | --- | --- | --- | --- |
|  | | Levene's Test for Equality of Variances | | t-test for Equality of Means | | | | | | |
|  |  | F | Sig. | t | df | Sig. (2-tailed) | Mean Difference | Std. Error Difference | 95% Confidence Interval of the Difference | |
|  |  |  |  |  |  |  |  |  | Lower | Upper |
| miR132 | Equal variances assumed | .279 | **.609** | 7.147 | 10 | **.000** | .488650565000000 | .068374947861112 | .336301687168120 | .640999442831880 |
|  | Equal variances not assumed |  |  | 7.147 | 9.784 | .000 | .488650565000000 | .068374947861112 | .335844182005118 | .641456947994883 |

**③miR-137**

| **Independent Samples Test** | | | | | | | | | | |
| --- | --- | --- | --- | --- | --- | --- | --- | --- | --- | --- |
|  | | Levene's Test for Equality of Variances | | t-test for Equality of Means | | | | | | |
|  |  | F | Sig. | t | df | Sig. (2-tailed) | Mean Difference | Std. Error Difference | 95% Confidence Interval of the Difference | |
|  |  |  |  |  |  |  |  |  | Lower | Upper |
| miR137 | Equal variances assumed | .014 | **.909** | .998 | 10 | **.342** | .034004404833333 | .034087228702558 | -.041946673795378 | .109955483462045 |
|  | Equal variances not assumed |  |  | .998 | 9.983 | .342 | .034004404833333 | .034087228702558 | -.041964233324452 | .109973042991118 |

**④miR-212**

| **Independent Samples Test** | | | | | | | | | | |
| --- | --- | --- | --- | --- | --- | --- | --- | --- | --- | --- |
|  | | Levene's Test for Equality of Variances | | t-test for Equality of Means | | | | | | |
|  |  | F | Sig. | t | df | Sig. (2-tailed) | Mean Difference | Std. Error Difference | 95% Confidence Interval of the Difference | |
|  |  |  |  |  |  |  |  |  | Lower | Upper |
| miR212 | Equal variances assumed | 3.183 | **.105** | 3.731 | 10 | **.004** | .117515903666667 | .031501232572621 | .047326783486155 | .187705023847178 |
|  | Equal variances not assumed |  |  | 3.731 | 8.635 | .005 | .117515903666667 | .031501232572621 | .045792967169513 | .189238840163821 |

**⑤miR-34a**

| **Independent Samples Test** | | | | | | | | | | |
| --- | --- | --- | --- | --- | --- | --- | --- | --- | --- | --- |
|  | | Levene's Test for Equality of Variances | | t-test for Equality of Means | | | | | | |
|  |  | F | Sig. | t | df | Sig. (2-tailed) | Mean Difference | Std. Error Difference | 95% Confidence Interval of the Difference | |
|  |  |  |  |  |  |  |  |  | Lower | Upper |
| miR34a | Equal variances assumed | .316 | **.587** | -3.171 | 10 | **.010** | -.093692379166667 | .029542514791637 | -.159517204159291 | -.027867554174042 |
|  | Equal variances not assumed |  |  | -3.171 | 9.631 | .010 | -.093692379166667 | .029542514791637 | -.159860230216789 | -.027524528116544 |

**⑥miR-9**

| **Independent Samples Test** | | | | | | | | | | |
| --- | --- | --- | --- | --- | --- | --- | --- | --- | --- | --- |
|  | | Levene's Test for Equality of Variances | | t-test for Equality of Means | | | | | | |
|  |  | F | Sig. | t | df | Sig. (2-tailed) | Mean Difference | Std. Error Difference | 95% Confidence Interval of the Difference | |
|  |  |  |  |  |  |  |  |  | Lower | Upper |
| miR9 | Equal variances assumed | .000 | **.992** | .796 | 10 | **.444** | .178302163500001 | .223965082768050 | -.320723138903814 | .677327465903815 |
|  | Equal variances not assumed |  |  | .796 | 9.874 | .445 | .178302163500001 | .223965082768050 | -.321586860520922 | .678191187520923 |

**Results**: MiR-124: The data were normally distributed (p > 0.05) with homogeneous variance (p = 0.49). An independent‑samples t‑test revealed no significant difference between the EE group (4.99 ± 0.32) and the SH group (4.60 ± 0.19), t(10) = 1.043, p = 0.321, 95% CI [–0.44, 1.21].

MiR-132: The data were normally distributed (p > 0.05) with homogeneous variance (p = 0.609). An independent‑samples t‑test showed that the EE group (1.61 ± 0.05) had significantly higher miR‑132 levels than the SH group (1.12 ± 0.05), t(10) = 7.147, p < 0.0001, 95% CI [0.34, 0.64].

MiR-137: The data were normally distributed (p > 0.05) with homogeneous variance (p = 0.909). An independent‑samples t‑test revealed no significant difference between the EE group (0.45 ± 0.02) and the SH group (0.41 ± 0.02), t(10) = 0.998, p = 0.342, 95% CI [0.04, 0.11].

MiR-212: The data were normally distributed (p > 0.05) with homogeneous variance (p = 0.105). An independent‑samples t‑test showed that the EE group (0.28 ± 0.03) had significantly higher miR‑212 levels than the SH group (0.17 ± 0.02), t(10) = 3.731, p = 0.004, 95% CI [0.05, 0.19].

MiR-34a: The data were normally distributed (p > 0.05) with homogeneous variance (p = 0.587). An independent‑samples t‑test showed that the EE group (0.16 ± 0.02) had significantly lower miR‑34a levels than the SH group (0.26 ± 0.02), t(10) = –3.171, p = 0.01, 95% CI [–0.16, –0.03].

MiR-9: The data were normally distributed (p > 0.05) with homogeneous variance (p = 0.992). An independent‑samples t‑test revealed no significant difference between the EE group (2.94 ± 0.15) and the SH group (2.76 ± 0.17), t(10) = 0.769, p = 0.444, 95% CI [–0.32, 0.68].

**Figure 3C-1: Paired sample t-test**

| **Paired Samples Test** | | | | | | | | | |
| --- | --- | --- | --- | --- | --- | --- | --- | --- | --- |
|  | | Paired Differences | | | | | t | df | Sig. (2-tailed) |
|  |  | Mean | Std. Deviation | Std. Error Mean | 95% Confidence Interval of the Difference | |  |  |  |
|  |  |  |  |  | Lower | Upper |  |  |  |
| Pair 1 | MD - Con | .257796874714286 | .184440056477721 | .069711788748394 | .087218272661334 | .428375476767238 | 3.698 | 6 | **.010** |

**结果： Shapiro-Wilk检验两组数据差值符合正态性（p =0.788），采用配对样本T检验比较发现MD组（M =0.72，SEM =0.06）的HDAC3表达显著高于Con组（M =0.16，SEM =0.05 ), t(6) = 3.698, p=0.01,95% CI [0.090, -0.43] 。**

**Results**: The difference between the two groups was normally distributed as assessed by Shapiro–Wilk test (p = 0.788). A paired‑samples t‑test revealed that HDAC3 expression was significantly higher in the MD group (0.72 ± 0.06) than in the Con group (0.16 ± 0.05), t(6) = 3.698, p = 0.01, 95% CI [0.090, 0.43]

**Figure 3C-2: one-way ANOVA**

| **ANOVA** | | | | | |
| --- | --- | --- | --- | --- | --- |
| HDAC3 | | | | | |
|  | Sum of Squares | df | Mean Square | F | Sig. |
| Between Groups | .413 | 3 | .138 | 3.589 | **.032** |
| Within Groups | .767 | 20 | .038 |  |  |
| Total | 1.180 | 23 |  |  |  |

| **Multiple Comparisons** | | | | | | |
| --- | --- | --- | --- | --- | --- | --- |
| Dependent Variable: HDAC3 | | | | | | |
| LSD | | | | | | |
| (I) Group | (J) Group | Mean Difference (I-J) | Std. Error | Sig. | 95% Confidence Interval | |
|  |  |  |  |  | Lower Bound | Upper Bound |
| SH-Con | SH-MD | -.304643273666667^*^ | .113086452809908 | .014 | -.540537480609080 | -.068749066724253 |
|  | EE-Con | -.060922547833333 | .113086452809908 | .596 | -.296816754775747 | .174971659109080 |
|  | EE-MD | .028142540208471 | .113086452809908 | .806 | -.207751666733943 | .264036747150884 |
| SH-MD | SH-Con | .304643273666667^*^ | .113086452809908 | **.014** | .068749066724253 | .540537480609080 |
|  | EE-Con | .243720725833333^*^ | .113086452809908 | .044 | .007826518890920 | .479614932775747 |
|  | EE-MD | .332785813875137^*^ | .113086452809908 | .008 | .096891606932724 | .568680020817551 |
| EE-Con | SH-Con | .060922547833333 | .113086452809908 | .596 | -.174971659109080 | .296816754775747 |
|  | SH-MD | -.243720725833333^*^ | .113086452809908 | .044 | -.479614932775747 | -.007826518890920 |
|  | EE-MD | .089065088041804 | .113086452809908 | .440 | -.146829118900610 | .324959294984217 |
| EE-MD | SH-Con | -.028142540208471 | .113086452809908 | .806 | -.264036747150884 | .207751666733943 |
|  | SH-MD | -.332785813875137^*^ | .113086452809908 | **.008** | -.568680020817551 | -.096891606932724 |
|  | EE-Con | -.089065088041804 | .113086452809908 | **.440** | -.324959294984217 | .146829118900610 |
| *. The mean difference is significant at the 0.05 level. | | | | | | |

**Results**: The data were normally distributed (p > 0.05) with homogeneous variance (Levene's F(3, 20) = 3.068, p = 0.051), meeting the assumptions for ANOVA. A one‑way ANOVA revealed a significant difference in HDAC3 levels among the four groups (SH‑Con: 0.51 ± 0.06; SH‑MD: 0.82 ± 0.14; EE‑Con: 0.57 ± 0.03; EE‑MD: 0.48 ± 0.10), F(3, 20) = 3.589, p = 0.032.

Post‑hoc LSD tests showed that the SH‑MD group had significantly higher HDAC3 levels than the SH‑Con group (mean difference = 0.30, p = 0.014, 95% CI [0.07, 0.54]), and the EE‑MD group had significantly lower HDAC3 levels than the SH‑MD group (mean difference = –0.33, p = 0.008, 95% CI [–0.57, –0.10]). No significant difference was observed between the EE‑MD and EE‑Con groups (mean difference = –0.09, p = 0.44, 95% CI [–0.32, 0.15]).

**Figure 3D-1: Paired sample t-test**

| **Paired Samples Test** | | | | | | | | | |
| --- | --- | --- | --- | --- | --- | --- | --- | --- | --- |
|  | | Paired Differences | | | | | t | df | Sig. (2-tailed) |
|  |  | Mean | Std. Deviation | Std. Error Mean | 95% Confidence Interval of the Difference | |  |  |  |
|  |  |  |  |  | Lower | Upper |  |  |  |
| Pair 1 | MD - Con | -.640463413000000 | .246775120121451 | .100745520918597 | -.899438019092915 | -.381488806907084 | -6.357 | 5 | **.001** |

**Results**: The difference between the two groups was normally distributed as assessed by the Shapiro–Wilk test (p = 0.364). A paired‑samples t‑test revealed that miR‑132 expression was significantly lower in the MD group (0.38 ± 0.09) than in the SH group (1.02 ± 0.01), t(5) = –6.357, p = 0.001, 95% CI [–0.90, –0.38].

**Figure 3D-2: one-way ANOVA**

| **ANOVA** | | | | | |
| --- | --- | --- | --- | --- | --- |
| miR132 | | | | | |
|  | Sum of Squares | df | Mean Square | F | Sig. |
| Between Groups | 2.323 | 3 | .774 | 15.757 | **.000** |
| Within Groups | 2.949 | 60 | .049 |  |  |
| Total | 5.273 | 63 |  |  |  |

| **Multiple Comparisons** | | | | | | |
| --- | --- | --- | --- | --- | --- | --- |
| Dependent Variable: miR132 | | | | | | |
| LSD | | | | | | |
| (I) Group | (J) Group | Mean Difference (I-J) | Std. Error | Sig. | 95% Confidence Interval | |
|  |  |  |  |  | Lower Bound | Upper Bound |
| SH-Con | SH-MD | .430421842066667^*^ | .080954261540129 | .000 | .268489209025204 | .592354475108130 |
|  | EE-Con | .071885737556863 | .078537169687960 | .364 | -.085211991917098 | .228983467030824 |
|  | EE-MD | -.075279294384314 | .078537169687960 | .342 | -.232377023858275 | .081818435089648 |
| SH-MD | SH-Con | -.430421842066667^*^ | .080954261540129 | **.000** | -.592354475108129 | -.268489209025204 |
|  | EE-Con | -.358536104509804^*^ | .078537169687960 | .000 | -.515633833983765 | -.201438375035843 |
|  | EE-MD | -.505701136450980^*^ | .078537169687960 | .000 | -.662798865924941 | -.348603406977019 |
| EE-Con | SH-Con | -.071885737556863 | .078537169687960 | .364 | -.228983467030824 | .085211991917098 |
|  | SH-MD | .358536104509804^*^ | .078537169687960 | .000 | .201438375035843 | .515633833983765 |
|  | EE-MD | -.147165031941176 | .076043287564934 | .058 | -.299274254436089 | .004944190553736 |
| EE-MD | SH-Con | .075279294384314 | .078537169687960 | .342 | -.081818435089648 | .232377023858275 |
|  | SH-MD | .505701136450980^*^ | .078537169687960 | **.000** | .348603406977019 | .662798865924941 |
|  | EE-Con | .147165031941176 | .076043287564934 | **.058** | -.004944190553736 | .299274254436089 |
| *. The mean difference is significant at the 0.05 level. | | | | | | |

**Results:** The data were normally distributed (p > 0.05) with homogeneous variance (Levene's F(3, 60) = 2.168, p = 0.101), meeting the assumptions for ANOVA. A one‑way ANOVA revealed a significant difference in miR‑132 levels among the four groups (SH‑Con: 0.64 ± 0.06; SH‑MD: 0.21 ± 0.04; EE‑Con: 0.56 ± 0.06; EE‑MD: 0.71 ± 0.06), F(3, 60) = 15.75, p < 0.0001.

Post‑hoc LSD tests showed that the SH‑MD group had significantly lower miR‑132 levels than the SH‑Con group (mean difference = –0.43, p < 0.0001, 95% CI [–0.59, –0.27]), and the EE‑MD group had significantly higher miR‑132 levels than the SH‑MD group (mean difference = 0.51, p < 0.0001, 95% CI [0.35, 0.66]). No significant difference was observed between the EE‑MD and EE‑Con groups (mean difference = 0.15, p = 0.058, 95% CI [–0.005, 0.30]).

**Figure 4B: one-way ANOVA**

| **ANOVA** | | | | | |
| --- | --- | --- | --- | --- | --- |
| luciferase | | | | | |
|  | Sum of Squares | df | Mean Square | F | Sig. |
| Between Groups | 24.291 | 3 | 8.097 | 72.154 | **.000** |
| Within Groups | .898 | 8 | .112 |  |  |
| Total | 25.189 | 11 |  |  |  |

| **Multiple Comparisons** | | | | | | |
| --- | --- | --- | --- | --- | --- | --- |
| Dependent Variable: luciferase | | | | | | |
| LSD | | | | | | |
| (I) Group | (J) Group | Mean Difference (I-J) | Std. Error | Sig. | 95% Confidence Interval | |
|  |  |  |  |  | Lower Bound | Upper Bound |
| WT+NC | WT+miR-132 mimics | 3.096540000000000^*^ | .273518438766424 | .000 | 2.465805349151413 | 3.727274650848588 |
|  | Mut+NC | -.298776000000000 | .273518438766424 | .306 | -.929510650848588 | .331958650848588 |
|  | Mut+miR-132 mimics | -.238349333333334 | .273518438766424 | .409 | -.869083984181921 | .392385317515254 |
| WT+miR-132 mimics | WT+NC | -3.096540000000000^*^ | .273518438766424 | **.000** | -3.727274650848588 | -2.465805349151412 |
|  | Mut+NC | -3.395316000000000^*^ | .273518438766424 | .000 | -4.026050650848588 | -2.764581349151412 |
|  | Mut+miR-132 mimics | -3.334889333333334^*^ | .273518438766424 | .000 | -3.965623984181921 | -2.704154682484746 |
| Mut+NC | WT+NC | .298776000000000 | .273518438766424 | .306 | -.331958650848588 | .929510650848588 |
|  | WT+miR-132 mimics | 3.395316000000000^*^ | .273518438766424 | **.000** | 2.764581349151413 | 4.026050650848588 |
|  | Mut+miR-132 mimics | .060426666666666 | .273518438766424 | .831 | -.570307984181921 | .691161317515254 |
| Mut+miR-132 mimics | WT+NC | .238349333333334 | .273518438766424 | .409 | -.392385317515254 | .869083984181922 |
|  | WT+miR-132 mimics | 3.334889333333334^*^ | .273518438766424 | **.000** | 2.704154682484746 | 3.965623984181922 |
|  | Mut+NC | -.060426666666666 | .273518438766424 | .831 | -.691161317515254 | .570307984181921 |
| *. The mean difference is significant at the 0.05 level. | | | | | | |

**Results:** The data were normally distributed (p > 0.05) with homogeneous variance (Levene's F(3, 8) = 0.628, p = 0.617), meeting the assumptions for ANOVA. A one-way ANOVA revealed a significant difference in luciferase activity among the four groups (WT+NC: 6.70 ± 0.15; WT+miR‑132 mimics: 3.61 ± 0.15; Mut+NC: 7.00 ± 0.17; Mut+miR‑132 mimics: 6.94 ± 0.28), F(3, 8) = 72.154, p < 0.0001.

Post‑hoc LSD tests showed that the WT+miR‑132 mimics group had significantly lower luciferase activity than the WT+NC group (mean difference = –3.10, p < 0.0001, 95% CI [–3.72, –2.47]), the Mut+NC group had significantly higher activity than the WT+miR‑132 mimics group (mean difference = 3.40, p < 0.0001, 95% CI [2.76, 4.03]), and the Mut+miR‑132 mimics group had significantly higher activity than the WT+miR‑132 mimics group (mean difference = 3.33, p < 0.0001, 95% CI [2.70, 3.97]).

**Figure 4C-1: one-way ANOVA**

| **ANOVA** | | | | | |
| --- | --- | --- | --- | --- | --- |
| miR132 | | | | | |
|  | Sum of Squares | df | Mean Square | F | Sig. |
| Between Groups | 1431.529 | 2 | 715.765 | 20155.101 | **.000** |
| Within Groups | .213 | 6 | .036 |  |  |
| Total | 1431.742 | 8 |  |  |  |

| **Multiple Comparisons** | | | | | | |
| --- | --- | --- | --- | --- | --- | --- |
| Dependent Variable: miR132 | | | | | | |
| LSD | | | | | | |
| (I) Group | (J) Group | Mean Difference (I-J) | Std. Error | Sig. | 95% Confidence Interval | |
|  |  |  |  |  | Lower Bound | Upper Bound |
| SH-SY5Y | +NC mimics | -.064200666666667 | .153867538732846 | .691 | -.440700970698559 | .312299637365226 |
|  | +miR-132 mimics | -26.785821000000000^*^ | .153867538732846 | .000 | -27.162321304031890 | -26.409320695968105 |
| +NC mimics | SH-SY5Y | .064200666666667 | .153867538732846 | **.691** | -.312299637365226 | .440700970698559 |
|  | +miR-132 mimics | -26.721620333333330^*^ | .153867538732846 | .000 | -27.098120637365227 | -26.345120029301440 |
| +miR-132 mimics | SH-SY5Y | 26.785821000000000^*^ | .153867538732846 | .000 | 26.409320695968105 | 27.162321304031890 |
|  | +NC mimics | 26.721620333333330^*^ | .153867538732846 | **.000** | 26.345120029301440 | 27.098120637365227 |
| *. The mean difference is significant at the 0.05 level. | | | | | | |

**Results:** The data were normally distributed (p > 0.05) with homogeneous variance (Levene's F(2, 6) = 3.293, p = 0.108), meeting the assumptions for ANOVA. A one‑way ANOVA revealed a significant difference in miR‑132 levels among the three groups (SH‑SY5Y: 1.14 ± 0.07; +NC mimics: 0.20 ± 0.04; +miR‑132 mimics: 27.92 ± 0.17), F(2, 6) = 20155.101, p < 0.0001.

Post‑hoc LSD tests showed that the +miR‑132 mimics group had significantly higher miR‑132 levels than the +NC mimics group (mean difference = 26.72, p < 0.0001, 95% CI [26.35, 27.10]), while no significant difference was observed between the +NC mimics and SH‑SY5Y groups (mean difference = 0.06, p = 0.691, 95% CI [–0.31, 0.44]).

**Figure 4C-2: one-way ANOVA**

| **ANOVA** | | | | | |
| --- | --- | --- | --- | --- | --- |
| HDAC3 | | | | | |
|  | Sum of Squares | df | Mean Square | F | Sig. |
| Between Groups | .708 | 2 | .354 | 621.493 | **.000** |
| Within Groups | .003 | 6 | .001 |  |  |
| Total | .711 | 8 |  |  |  |

| **Multiple Comparisons** | | | | | | |
| --- | --- | --- | --- | --- | --- | --- |
| Dependent Variable: HDAC3 | | | | | | |
| LSD | | | | | | |
| (I) Group | (J) Group | Mean Difference (I-J) | Std. Error | Sig. | 95% Confidence Interval | |
|  |  |  |  |  | Lower Bound | Upper Bound |
| SH-SY5Y | +NC mimics | -.006720833333333 | .019483016826381 | .742 | -.054394058101859 | .040952391435193 |
|  | +miR-132 mimics | .591478433333333^*^ | .019483016826381 | .000 | .543805208564807 | .639151658101859 |
| +NC mimics | SH-SY5Y | .006720833333333 | .019483016826381 | **.742** | -.040952391435193 | .054394058101859 |
|  | +miR-132 mimics | .598199266666667^*^ | .019483016826381 | .000 | .550526041898141 | .645872491435193 |
| +miR-132 mimics | SH-SY5Y | -.591478433333333^*^ | .019483016826381 | .000 | -.639151658101859 | -.543805208564807 |
|  | +NC mimics | -.598199266666667^*^ | .019483016826381 | **.000** | -.645872491435193 | -.550526041898141 |
| *. The mean difference is significant at the 0.05 level. | | | | | | |

**Results:** The data were normally distributed (p > 0.05) with homogeneous variance (Levene's F(2, 6) = 0.110, p = 0.898), meeting the assumptions for ANOVA. A one‑way ANOVA revealed a significant difference in HDAC3 levels among the three groups (SH‑SY5Y: 0.98 ± 0.02; +NC mimics: 0.98 ± 0.01; +miR‑132 mimics: 0.38 ± 0.01), F(2, 6) = 621.493, p < 0.0001.

Post‑hoc LSD tests showed that the +miR‑132 mimics group had significantly lower HDAC3 levels than the +NC mimics group (mean difference = –0.60, p < 0.0001, 95% CI [–0.65, –0.55]), while no significant difference was observed between the +NC mimics and SH‑SY5Y groups (mean difference = 0.007, p = 0.742, 95% CI [–0.04, 0.05])

**Figure 4D-1: one-way ANOVA**

| **Test of Homogeneity of Variances** | | | | | |
| --- | --- | --- | --- | --- | --- |
|  | | Levene Statistic | df1 | df2 | Sig. |
| miR132 | Based on Mean | 4.894 | 6 | 14 | **.007** |
|  | Based on Median | 1.080 | 6 | 14 | .420 |
|  | Based on Median and with adjusted df | 1.080 | 6 | 3.778 | .497 |
|  | Based on trimmed mean | 4.447 | 6 | 14 | .010 |

| **Robust Tests of Equality of Means** | | | | |
| --- | --- | --- | --- | --- |
| miR132 | | | | |
|  | Statistic^a^ | df1 | df2 | Sig. |
| Welch | 101.374 | 6 | 5.893 | **.000** |
| a. Asymptotically F distributed. | | | | |

| **Multiple Comparisons** | | | | | | |
| --- | --- | --- | --- | --- | --- | --- |
| Dependent Variable: miR132 | | | | | | |
| Games-Howell | | | | | | |
| (I) Group | (J) Group | Mean Difference (I-J) | Std. Error | Sig. | 95% Confidence Interval | |
|  |  |  |  |  | Lower Bound | Upper Bound |
| Visual cortex neurons | +NC mimics | .006115000000000 | .073067652584589 | 1.000 | -.368232259922631 | .380462259922631 |
|  | +miR-132 mimics | -1.843405000000000^*^ | .121763163855996 | .003 | -2.565303680589558 | -1.121506319410441 |
|  | +miR-132 mimics+Empty vector | -1.847648000000000^*^ | .086823466433652 | .000 | -2.284909861302216 | -1.410386138697784 |
|  | +miR-132 mimics+HDAC3 overexpression vector | -1.582455666666666 | .269839697653584 | .093 | -3.728941336393258 | .564030003059926 |
|  | +Empty vector | -.103823333333333 | .060812644999941 | .661 | -.524056579250091 | .316409912583425 |
|  | +HDAC3 overexpression vector | -.026775333333333 | .087809912909775 | 1.000 | -.470351665880660 | .416800999213993 |
| +NC mimics | Visual cortex neurons | -.006115000000000 | .073067652584589 | **1.000** | -.380462259922631 | .368232259922631 |
|  | +miR-132 mimics | -1.849520000000000^*^ | .116787965531081 | .005 | -2.611612756398487 | -1.087427243601513 |
|  | +miR-132 mimics+Empty vector | -1.853763000000000^*^ | .079696142593116 | .000 | -2.278172583644988 | -1.429353416355012 |
|  | +miR-132 mimics+HDAC3 overexpression vector | -1.588570666666666 | .267631506460739 | .096 | -3.791498103075927 | .614356769742595 |
|  | +Empty vector | -.109938333333333 | .050113257852145 | .479 | -.426577278898398 | .206700612231731 |
|  | +HDAC3 overexpression vector | -.032890333333333 | .080769682591235 | .999 | -.465928211844362 | .400147545177695 |
| +miR-132 mimics | Visual cortex neurons | 1.843405000000000^*^ | .121763163855996 | .003 | 1.121506319410441 | 2.565303680589559 |
|  | +NC mimics | 1.849520000000000^*^ | .116787965531081 | **.005** | 1.087427243601513 | 2.611612756398487 |
|  | +miR-132 mimics+Empty vector | -.004243000000000 | .125852538163607 | 1.000 | -.709311388926218 | .700825388926217 |
|  | +miR-132 mimics+HDAC3 overexpression vector | .260949333333334 | .284805213204472 | .944 | -1.626447954796066 | 2.148346621462734 |
|  | +Empty vector | 1.739581666666667^*^ | .109538691020824 | .012 | .852184268840494 | 2.626979064492840 |
|  | +HDAC3 overexpression vector | 1.816629666666667^*^ | .126535085426489 | .003 | 1.113296930050534 | 2.519962403282799 |
| +miR-132 mimics+Empty vector | Visual cortex neurons | 1.847648000000000^*^ | .086823466433652 | .000 | 1.410386138697784 | 2.284909861302217 |
|  | +NC mimics | 1.853763000000000^*^ | .079696142593116 | .000 | 1.429353416355013 | 2.278172583644988 |
|  | +miR-132 mimics | .004243000000000 | .125852538163607 | 1.000 | -.700825388926217 | .709311388926218 |
|  | +miR-132 mimics+HDAC3 overexpression vector | .265192333333334 | .271709506126999 | .925 | -1.837962235952309 | 2.368346902618978 |
|  | +Empty vector | 1.743824666666667^*^ | .068635057236728 | .003 | 1.247312966282172 | 2.240336367051163 |
|  | +HDAC3 overexpression vector | 1.820872666666667^*^ | .093397934105751 | .000 | 1.355006724159447 | 2.286738609173887 |
| +miR-132 mimics+HDAC3 overexpression vector | Visual cortex neurons | 1.582455666666666 | .269839697653584 | .093 | -.564030003059926 | 3.728941336393258 |
|  | +NC mimics | 1.588570666666666 | .267631506460739 | .096 | -.614356769742595 | 3.791498103075928 |
|  | +miR-132 mimics | -.260949333333334 | .284805213204472 | .944 | -2.148346621462734 | 1.626447954796066 |
|  | +miR-132 mimics+Empty vector | -.265192333333334 | .271709506126999 | .925 | -2.368346902618977 | 1.837962235952309 |
|  | +Empty vector | 1.478632333333333 | .264548519534907 | .116 | -.813914191176431 | 3.771178857843097 |
|  | +HDAC3 overexpression vector | 1.555680333333333 | .272026326302151 | .094 | -.540508259435031 | 3.651868926101697 |
| +Empty vector | Visual cortex neurons | .103823333333333 | .060812644999941 | .661 | -.316409912583425 | .524056579250091 |
|  | +NC mimics | .109938333333333 | .050113257852145 | .479 | -.206700612231731 | .426577278898398 |
|  | +miR-132 mimics | -1.739581666666667^*^ | .109538691020824 | .012 | -2.626979064492840 | -.852184268840493 |
|  | +miR-132 mimics+Empty vector | -1.743824666666667^*^ | .068635057236728 | .003 | -2.240336367051162 | -1.247312966282171 |
|  | +miR-132 mimics+HDAC3 overexpression vector | -1.478632333333333 | .264548519534907 | **.116** | -3.771178857843097 | .813914191176431 |
|  | +HDAC3 overexpression vector | .077048000000000 | .069878734702022 | .889 | -.431571048538681 | .585667048538680 |
| +HDAC3 overexpression vector | Visual cortex neurons | .026775333333333 | .087809912909775 | **1.000** | -.416800999213993 | .470351665880660 |
|  | +NC mimics | .032890333333333 | .080769682591235 | .999 | -.400147545177695 | .465928211844362 |
|  | +miR-132 mimics | -1.816629666666666^*^ | .126535085426489 | .003 | -2.519962403282799 | -1.113296930050534 |
|  | +miR-132 mimics+Empty vector | -1.820872666666667^*^ | .093397934105751 | .000 | -2.286738609173887 | -1.355006724159446 |
|  | +miR-132 mimics+HDAC3 overexpression vector | -1.555680333333333 | .272026326302151 | .094 | -3.651868926101696 | .540508259435031 |
|  | +Empty vector | -.077048000000000 | .069878734702022 | .889 | -.585667048538680 | .431571048538681 |
| *. The mean difference is significant at the 0.05 level. | | | | | | |

**Results:** The data were normally distributed (p > 0.05). Since Levene's test was significant (F(6, 14) = 4.894, p = 0.007), indicating unequal variances, Welch's ANOVA was used to compare miR‑132 levels among the seven groups. The groups were: Visual cortex neurons (1.10 ± 0.06), +NC mimics (0.10 ± 0.05), +miR‑132 mimics (2.95 ± 0.11), +miR‑132 mimics+Empty vector (2.95 ± 0.07), +miR‑132 mimics+HDAC3 overexpression vector (2.68 ± 0.26), +Empty vector (1.21 ± 0.02), and +HDAC3 overexpression vector (1.13 ± 0.07). Welch's ANOVA revealed a significant difference among the groups (Welch's F(6, 5.893) = 101.374, p < 0.0001).

Post‑hoc comparisons using the Games‑Howell test showed that the +miR‑132 mimics group had significantly higher miR‑132 levels than the +NC mimics group (mean difference = 1.85, p = 0.005, 95% CI [1.09, 2.61]), while no significant difference was observed between the +HDAC3 overexpression vector group and the Visual cortex neurons group (mean difference = 0.03, p = 1.00, 95% CI [–0.42, 0.47]).

**Figure 4D-2: one-way ANOVA**

| **Test of Homogeneity of Variances** | | | | | |
| --- | --- | --- | --- | --- | --- |
|  | | Levene Statistic | df1 | df2 | Sig. |
| HDAC3 | Based on Mean | 4.345 | 6 | 14 | **.011** |
|  | Based on Median | .934 | 6 | 14 | .501 |
|  | Based on Median and with adjusted df | .934 | 6 | 6.570 | .528 |
|  | Based on trimmed mean | 3.942 | 6 | 14 | .016 |

| **Robust Tests of Equality of Means** | | | | |
| --- | --- | --- | --- | --- |
| HDAC3 | | | | |
|  | Statistic^a^ | df1 | df2 | Sig. |
| Welch | 281.419 | 6 | 5.972 | **.000** |
| a. Asymptotically F distributed. | | | | |

| **Multiple Comparisons** | | | | | | |
| --- | --- | --- | --- | --- | --- | --- |
| Dependent Variable: HDAC3 | | | | | | |
| Games-Howell | | | | | | |
| (I) Group | (J) Group | Mean Difference (I-J) | Std. Error | Sig. | 95% Confidence Interval | |
|  |  |  |  |  | Lower Bound | Upper Bound |
| Visual cortex neurons | +NC mimics | -.236162764333333 | .108563774939284 | .482 | -.906032103521343 | .433706574854676 |
|  | +miR-132 mimics | .992662702666667^*^ | .097982084464019 | .037 | .142060033521233 | 1.843265371812100 |
|  | +miR-132 mimics+Empty vector | 1.023509526666667^*^ | .098407821375522 | .034 | .185933945661232 | 1.861085107672101 |
|  | +miR-132 mimics+HDAC3 overexpression vector | .632708073333333 | .098057051083119 | .088 | -.215547132792214 | 1.480963279458881 |
|  | +Empty vector | -.181661441000000 | .116568586515815 | .715 | -.817806902087412 | .454484020087412 |
|  | +HDAC3 overexpression vector | -.857685602000000^*^ | .126367054021117 | .019 | -1.501197964114429 | -.214173239885571 |
| +NC mimics | Visual cortex neurons | .236162764333334 | .108563774939284 | .482 | -.433706574854676 | .906032103521343 |
|  | +miR-132 mimics | 1.228825467000000^*^ | .047858483440602 | .005 | .829530553504802 | 1.628120380495198 |
|  | +miR-132 mimics+Empty vector | 1.259672291000000^*^ | .048724171303338 | .004 | .882048582003191 | 1.637295999996810 |
|  | +miR-132 mimics+HDAC3 overexpression vector | .868870837666667^*^ | .048011778017875 | .010 | .473789496194718 | 1.263952179138615 |
|  | +Empty vector | .054501323333333 | .079234972856784 | .986 | -.358843800912586 | .467846447579253 |
|  | +HDAC3 overexpression vector | -.621522837666667^*^ | .093051479855488 | .031 | -1.149119487029578 | -.093926188303755 |
| +miR-132 mimics | Visual cortex neurons | -.992662702666667^*^ | .097982084464019 | .037 | -1.843265371812100 | -.142060033521233 |
|  | +NC mimics | -1.228825467000000^*^ | .047858483440602 | **.005** | -1.628120380495197 | -.829530553504802 |
|  | +miR-132 mimics+Empty vector | .030846824000000 | .013727363768896 | .443 | -.045428179706444 | .107121827706444 |
|  | +miR-132 mimics+HDAC3 overexpression vector | -.359954629333333^*^ | .010932816438794 | .000 | -.414926848896582 | -.304982409770085 |
|  | +Empty vector | -1.174324143666667^*^ | .063973248865927 | .011 | -1.720359880112495 | -.628288407220839 |
|  | +HDAC3 overexpression vector | -1.850348304666667^*^ | .080454791965071 | .007 | -2.544534926954902 | -1.156161682378432 |
| +miR-132 mimics+Empty vector | Visual cortex neurons | -1.023509526666666^*^ | .098407821375522 | .034 | -1.861085107672101 | -.185933945661232 |
|  | +NC mimics | -1.259672291000000^*^ | .048724171303338 | .004 | -1.637295999996809 | -.882048582003190 |
|  | +miR-132 mimics | -.030846824000000 | .013727363768896 | .443 | -.107121827706444 | .045428179706444 |
|  | +miR-132 mimics+HDAC3 overexpression vector | -.390801453333333^*^ | .014252610541476 | .000 | -.466500357734190 | -.315102548932476 |
|  | +Empty vector | -1.205170967666667^*^ | .064623424564352 | .009 | -1.732933830188822 | -.677408105144511 |
|  | +HDAC3 overexpression vector | -1.881195128666666^*^ | .080972736041864 | .006 | -2.560010937461117 | -1.202379319872216 |
| +miR-132 mimics+HDAC3 overexpression vector | Visual cortex neurons | -.632708073333333 | .098057051083119 | .088 | -1.480963279458881 | .215547132792215 |
|  | +NC mimics | -.868870837666667^*^ | .048011778017875 | .010 | -1.263952179138615 | -.473789496194718 |
|  | +miR-132 mimics | .359954629333333^*^ | .010932816438794 | .000 | .304982409770085 | .414926848896582 |
|  | +miR-132 mimics+Empty vector | .390801453333333^*^ | .014252610541476 | **.000** | .315102548932476 | .466500357734190 |
|  | +Empty vector | -.814369514333333^*^ | .064088009499947 | .022 | -1.357003317619473 | -.271735711047194 |
|  | +HDAC3 overexpression vector | -1.490393675333333^*^ | .080546073407375 | .011 | -2.181777572306799 | -.799009778359868 |
| +Empty vector | Visual cortex neurons | .181661441000000 | .116568586515815 | .715 | -.454484020087411 | .817806902087412 |
|  | +NC mimics | -.054501323333333 | .079234972856784 | .986 | -.467846447579253 | .358843800912586 |
|  | +miR-132 mimics | 1.174324143666667^*^ | .063973248865927 | .011 | .628288407220839 | 1.720359880112495 |
|  | +miR-132 mimics+Empty vector | 1.205170967666667^*^ | .064623424564352 | .009 | .677408105144511 | 1.732933830188823 |
|  | +miR-132 mimics+HDAC3 overexpression vector | .814369514333333^*^ | .064088009499947 | **.022** | .271735711047194 | 1.357003317619473 |
|  | +HDAC3 overexpression vector | -.676024161000000^*^ | .102277661473675 | .021 | -1.200701129126574 | -.151347192873426 |
| +HDAC3 overexpression vector | Visual cortex neurons | .857685602000000^*^ | .126367054021117 | .019 | .214173239885571 | 1.501197964114430 |
|  | +NC mimics | .621522837666667^*^ | .093051479855488 | .031 | .093926188303755 | 1.149119487029578 |
|  | +miR-132 mimics | 1.850348304666667^*^ | .080454791965071 | .007 | 1.156161682378432 | 2.544534926954902 |
|  | +miR-132 mimics+Empty vector | 1.881195128666667^*^ | .080972736041864 | .006 | 1.202379319872216 | 2.560010937461117 |
|  | +miR-132 mimics+HDAC3 overexpression vector | 1.490393675333333^*^ | .080546073407375 | .011 | .799009778359868 | 2.181777572306799 |
|  | +Empty vector | .676024161000000^*^ | .102277661473675 | **.021** | .151347192873426 | 1.200701129126574 |
| *. The mean difference is significant at the 0.05 level. | | | | | | |

**Results:** The data were normally distributed (p > 0.05). Since Levene's test was significant (F(6, 14) = 4.345, p = 0.011), indicating unequal variances, Welch's ANOVA was used to compare HDAC3 levels among the seven groups. The groups were: Visual cortex neurons (1.14 ± 0.10), +NC mimics (1.38 ± 0.05), +miR-132 mimics (0.51 ± 0.01), +miR-132 mimics+Empty vector (0.12 ± 0.02), +miR-132 mimics+HDAC3 overexpression vector (0.51 ± 0.06), +Empty vector (1.32 ± 0.01), and +HDAC3 overexpression vector (2.00 ± 0.14). Welch's ANOVA revealed a significant difference among the groups (Welch's F(6, 5.972) = 281.419, p < 0.0001).

Post-hoc comparisons using the Games-Howell test showed that:

The +miR-132 mimics group had significantly lower HDAC3 levels than the +NC mimics group (mean difference = –1.22, p = 0.005, 95% CI [–1.63, –0.83]).

The +miR-132 mimics+HDAC3 overexpression vector group had significantly higher HDAC3 levels than the +miR-132 mimics+Empty vector group (mean difference = 0.39, p < 0.0001, 95% CI [0.32, 0.47]).

The +Empty vector group had significantly higher HDAC3 levels than the +miR-132 mimics+HDAC3 overexpression vector group (mean difference = 0.81, p = 0.022, 95% CI [0.27, 1.36]).

The +HDAC3 overexpression vector group had significantly higher HDAC3 levels than the +Empty vector group (mean difference = 0.68, p = 0.021, 95% CI [0.15, 1.20]).

**Figure 6: one-way ANOVA**

| **ANOVA** | | | | | |
| --- | --- | --- | --- | --- | --- |
| MAP2 | | | | | |
|  | Sum of Squares | df | Mean Square | F | Sig. |
| Between Groups | .032 | 6 | .005 | 101.733 | **.000** |
| Within Groups | .001 | 14 | .000 |  |  |
| Total | .032 | 20 |  |  |  |

| **Multiple Comparisons** | | | | | | |
| --- | --- | --- | --- | --- | --- | --- |
| Dependent Variable: MAP2 | | | | | | |
| LSD | | | | | | |
| (I) Group | (J) Group | Mean Difference (I-J) | Std. Error | Sig. | 95% Confidence Interval | |
|  |  |  |  |  | Lower Bound | Upper Bound |
| Visual cortex neurons | +NC mimics | .002516299333333 | .005884907363248 | .675 | -.010105571638990 | .015138170305657 |
|  | +miR-132 mimics | -.077680944666667^*^ | .005884907363248 | .000 | -.090302815638990 | -.065059073694343 |
|  | +miR-132 mimics+Empty vector | -.083107935333333^*^ | .005884907363248 | .000 | -.095729806305657 | -.070486064361010 |
|  | +miR-132 mimics+HDAC3 overexpression vector | -.023246953000000^*^ | .005884907363248 | .001 | -.035868823972323 | -.010625082027677 |
|  | +Empty vector | .004099727000000 | .005884907363248 | .497 | -.008522143972323 | .016721597972323 |
|  | +HDAC3 overexpression vector | .022356159000000^*^ | .005884907363248 | .002 | .009734288027677 | .034978029972323 |
| +NC mimics | Visual cortex neurons | -.002516299333333 | .005884907363248 | .675 | -.015138170305657 | .010105571638990 |
|  | +miR-132 mimics | -.080197244000000^*^ | .005884907363248 | .000 | -.092819114972323 | -.067575373027677 |
|  | +miR-132 mimics+Empty vector | -.085624234666667^*^ | .005884907363248 | .000 | -.098246105638990 | -.073002363694343 |
|  | +miR-132 mimics+HDAC3 overexpression vector | -.025763252333333^*^ | .005884907363248 | .001 | -.038385123305657 | -.013141381361010 |
|  | +Empty vector | .001583427666667 | .005884907363248 | .792 | -.011038443305657 | .014205298638990 |
|  | +HDAC3 overexpression vector | .019839859666667^*^ | .005884907363248 | .005 | .007217988694343 | .032461730638990 |
| +miR-132 mimics | Visual cortex neurons | .077680944666667^*^ | .005884907363248 | .000 | .065059073694343 | .090302815638990 |
|  | +NC mimics | .080197244000000^*^ | .005884907363248 | **.000** | .067575373027677 | .092819114972323 |
|  | +miR-132 mimics+Empty vector | -.005426990666667 | .005884907363248 | .372 | -.018048861638990 | .007194880305657 |
|  | +miR-132 mimics+HDAC3 overexpression vector | .054433991666667^*^ | .005884907363248 | .000 | .041812120694343 | .067055862638990 |
|  | +Empty vector | .081780671666667^*^ | .005884907363248 | .000 | .069158800694343 | .094402542638990 |
|  | +HDAC3 overexpression vector | .100037103666667^*^ | .005884907363248 | .000 | .087415232694343 | .112658974638990 |
| +miR-132 mimics+Empty vector | Visual cortex neurons | .083107935333333^*^ | .005884907363248 | .000 | .070486064361010 | .095729806305657 |
|  | +NC mimics | .085624234666667^*^ | .005884907363248 | .000 | .073002363694343 | .098246105638990 |
|  | +miR-132 mimics | .005426990666667 | .005884907363248 | .372 | -.007194880305657 | .018048861638990 |
|  | +miR-132 mimics+HDAC3 overexpression vector | .059860982333333^*^ | .005884907363248 | .000 | .047239111361010 | .072482853305657 |
|  | +Empty vector | .087207662333333^*^ | .005884907363248 | .000 | .074585791361010 | .099829533305657 |
|  | +HDAC3 overexpression vector | .105464094333333^*^ | .005884907363248 | .000 | .092842223361010 | .118085965305657 |
| +miR-132 mimics+HDAC3 overexpression vector | Visual cortex neurons | .023246953000000^*^ | .005884907363248 | .001 | .010625082027677 | .035868823972323 |
|  | +NC mimics | .025763252333333^*^ | .005884907363248 | .001 | .013141381361010 | .038385123305657 |
|  | +miR-132 mimics | -.054433991666667^*^ | .005884907363248 | .000 | -.067055862638990 | -.041812120694343 |
|  | +miR-132 mimics+Empty vector | -.059860982333333^*^ | .005884907363248 | **.000** | -.072482853305657 | -.047239111361010 |
|  | +Empty vector | .027346680000000^*^ | .005884907363248 | .000 | .014724809027677 | .039968550972323 |
|  | +HDAC3 overexpression vector | .045603112000000^*^ | .005884907363248 | .000 | .032981241027677 | .058224982972323 |
| +Empty vector | Visual cortex neurons | -.004099727000000 | .005884907363248 | .497 | -.016721597972323 | .008522143972323 |
|  | +NC mimics | -.001583427666667 | .005884907363248 | .792 | -.014205298638990 | .011038443305657 |
|  | +miR-132 mimics | -.081780671666667^*^ | .005884907363248 | .000 | -.094402542638990 | -.069158800694343 |
|  | +miR-132 mimics+Empty vector | -.087207662333333^*^ | .005884907363248 | .000 | -.099829533305657 | -.074585791361010 |
|  | +miR-132 mimics+HDAC3 overexpression vector | -.027346680000000^*^ | .005884907363248 | **.000** | -.039968550972323 | -.014724809027677 |
|  | +HDAC3 overexpression vector | .018256432000000^*^ | .005884907363248 | .008 | .005634561027677 | .030878302972323 |
| +HDAC3 overexpression vector | Visual cortex neurons | -.022356159000000^*^ | .005884907363248 | .002 | -.034978029972323 | -.009734288027677 |
|  | +NC mimics | -.019839859666667^*^ | .005884907363248 | .005 | -.032461730638990 | -.007217988694343 |
|  | +miR-132 mimics | -.100037103666667^*^ | .005884907363248 | .000 | -.112658974638990 | -.087415232694343 |
|  | +miR-132 mimics+Empty vector | -.105464094333333^*^ | .005884907363248 | .000 | -.118085965305657 | -.092842223361010 |
|  | +miR-132 mimics+HDAC3 overexpression vector | -.045603112000000^*^ | .005884907363248 | .000 | -.058224982972323 | -.032981241027677 |
|  | +Empty vector | -.018256432000000^*^ | .005884907363248 | **.008** | -.030878302972323 | -.005634561027677 |
| *. The mean difference is significant at the 0.05 level. | | | | | | |

**Results:** The data were normally distributed (p > 0.05) with homogeneous variance (Levene's F(6, 14) = 1.859, p = 0.159), meeting the assumptions for ANOVA. A one-way ANOVA revealed a significant difference in MAP‑2 levels among the seven groups (Visual cortex neurons: 0.05 ± 0.001; +NC mimics: 0.05 ± 0.003; +miR‑132 mimics: 0.13 ± 0.006; +miR‑132 mimics+Empty vector: 0.13 ± 0.007; +miR‑132 mimics+HDAC3 overexpression vector: 0.07 ± 0.003; +Empty vector: 0.05 ± 0.002; +HDAC3 overexpression vector: 0.03 ± 0.002), F(6, 14) = 101.733, p < 0.0001.

Post‑hoc LSD tests showed that:

The +miR‑132 mimics group had significantly higher MAP‑2 levels than the +NC mimics group (mean difference = 0.08, p < 0.0001, 95% CI [0.07, 0.09]).

The +miR‑132 mimics+HDAC3 overexpression vector group had significantly lower MAP‑2 levels than the +miR‑132 mimics+Empty vector group (mean difference = –0.06, p < 0.0001, 95% CI [–0.07, –0.05]).

The +Empty vector group had significantly lower MAP‑2 levels than the +miR‑132 mimics+HDAC3 overexpression vector group (mean difference = –0.03, p < 0.0001, 95% CI [–0.04, –0.01]).

The +HDAC3 overexpression vector group had significantly lower MAP‑2 levels than the +Empty vector group (mean difference = –0.02, p = 0.008, 95% CI [–0.03, –0.01]).
